# Supplementary material for: Cellular heterogeneity results from indirect effects under metabolic tradeoffs
Source: R Soc Open Sci. 2019 Sep 25;6(9):190281. doi: 10.1098/rsos.190281 (PMC6774940; doi:10.1098/rsos.190281)
Supplement: Cellular heterogeneity results from indirect effects under metabolic tradeoffs. Supplementary Material. [file rsos190281supp1.pdf]

Cellular heterogeneity results from indirect effects under metabolic tradeoffs
Supplementary Material

Aina Ollé-Vila[1,2] and Ricard Solé[1,2,3*]
[1]ICREA-Complex Systems Lab, Universitat Pompeu Fabra, 08003 Barcelona
[2]Institut de Biologia Evolutiva (CSIC-UPF), Psg Maritim Barceloneta, 37, 08003 Barcelona and
[3]Santa Fe Institute, 399 Hyde Park Road, Santa Fe NM 87501, USA

I. BASIC MODEL OF RESOURCES AND ONE CONSUMER

The well-known model of one resource and one consumer is useful for comparison in this work. The equations defining this two-dimensional system are:

$$\dot{R} = \mu - \delta_R - \eta RS \tag{1}$$

$$\dot{S} = \rho RS - \delta_S S \tag{2}$$

Assuming R rapidly reaches equilibrium due to its reaction time scale, the system can be reduced to one dimension:

$$\dot{S} = \rho \frac{\mu}{\delta_R + \eta S} S - \delta_S S = f(S) \tag{3}$$

The fixed points of this equation are:

$$S^{*(1)} = 0$$

$$S^{*(2)} = \frac{\rho\mu - \delta_S \delta_R}{\delta_S \eta}$$

Using the linearization about $S^{*(1)}$ we can assess the stability of the system. Let $\xi(t) = S(t) - S^{*(1)}$ be a small perturbation away from $S^{*(1)}$. To see whether the perturbation grows or decays we need to derive a differential equation for ξ, which reads:

$$\dot{\xi} = \frac{d}{dt}(S - S^{*(1)}) = \dot{S} \tag{4}$$

Thus, $\dot{\xi} = \dot{S} = f(S) = f(S^{*(1)} + \xi)$. Using the Taylor expansion and ignoring higher order terms we obtain $f(S^{*(1)} + \xi) = f(S^{*(1)}) + \xi f'(S^{*(1)})$. As $f(S^{*(1)}) = 0$, we are left with the following expression:

$$\begin{aligned}\dot{\xi} &= \xi f'(S^{*(1)}) \\ &= \xi\Big(\frac{\mu\rho}{\delta_R + \eta S} - \frac{\mu\rho\eta S}{(\delta_R + \eta S)^2} - \delta_S\Big) \\ &= \xi\Big(\frac{\mu\rho}{\delta_R} - \delta_S\Big)\end{aligned}$$

the perturbation $\xi(t)$ will decay exponentially if $\mu\rho/\delta_R < \delta_S$ (that is, $S^{*(1)}$ is stable and $S^{*(2)}$ unstable under this condition) and will grow exponentially if $\mu\rho/\delta_R > \delta_S$ (that is, $S^{*(1)}$ is unstable and $S^{*(2)}$ stable under this condition). We observe the presence of a transcritical bifurcation as a function of the parameter μ (see Figure S1a)) with a critical value at:

$$\mu_c = \frac{\delta_s \delta_R}{\rho}$$

* ricard.sole@upf.edu

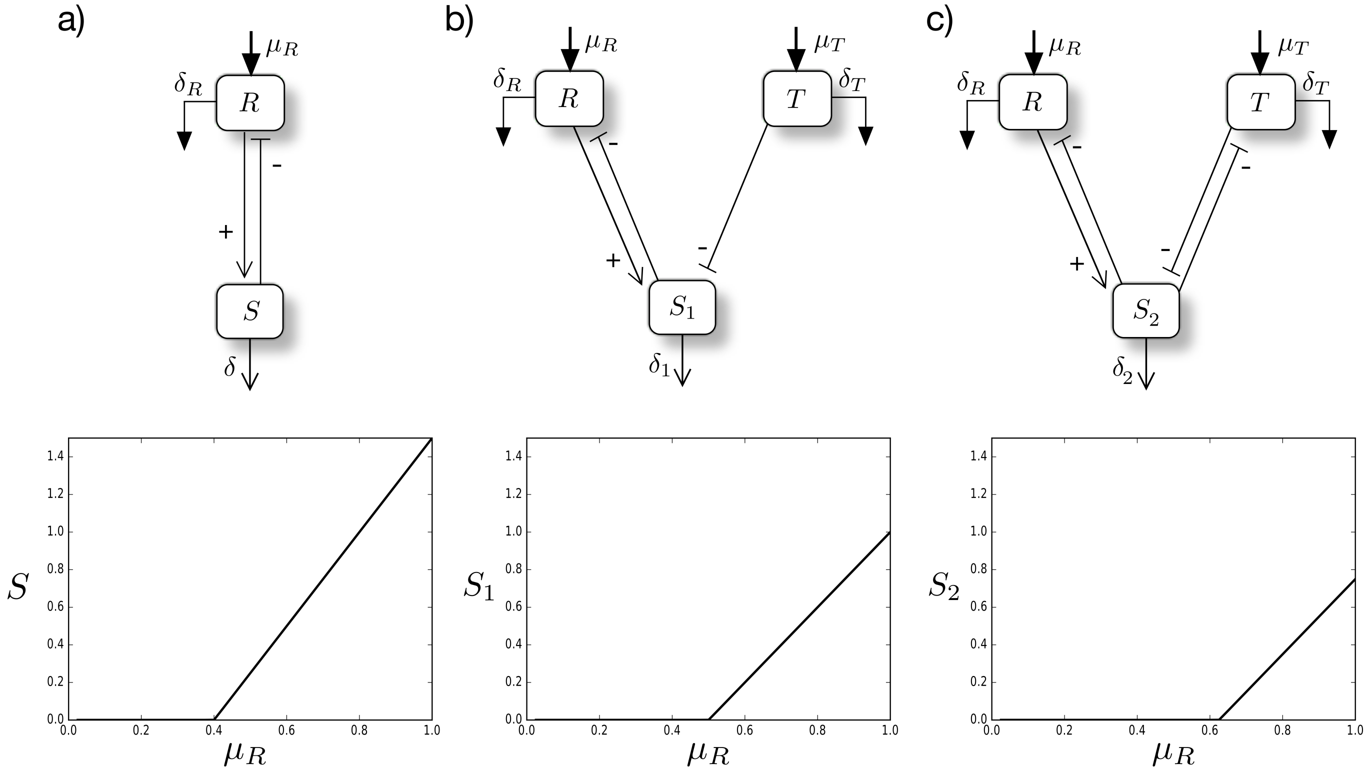

FIG. 1: Transcritical bifurcations associated to the simple models a) Resource-Consumer model, b) Resource-Toxic S_1 model and c) Resource-Toxic S_2 model. This allows to compare the fitness of S_1 and S_2 species under the same environment. Despite not degrading T, the lack of a metabolic tradeoff allows S_1 a better survival under these conditions.

II. SURVIVAL OF SPECIES 1 (S_1) WITH TOXIC AND RESOURCES

The region of parameters where only S_1 survives is equivalent to this three dimensional system:

$$\dot{T} = \mu_T - \delta_T T \tag{5}$$
$$\dot{R} = \mu_R - \delta_R R - \eta S_1 R \tag{6}$$
$$\dot{S}_1 = \rho R S_1 - \gamma_1 T S_1 - \delta_1 S_1 \tag{7}$$

Assuming that either T and R rapidly reach equilibrium due to their reaction time scale, the system can have its dimensionality reduced to one:

$$\dot{S}_1 = \rho\Big(\frac{\mu_R}{\delta_R + \eta S_1}\Big)S_1 - \gamma_1\Big(\frac{\mu_T}{\delta_T}\Big)S_1 - \delta_1 S_1 = g(S_1) \tag{8}$$

which has the following fixed points:

$$S_1^{*(1)} = 0$$
$$S_1^{*(2)} = \frac{\rho\mu_R\delta_T - \delta_R\gamma_1\mu_T - \delta_1\delta_R\delta_T}{\eta(\gamma_1\mu_T + \delta_1\delta_T)}$$

Using the linearization about $S_1^{*(1)}$ (see section I for a detailed derivation) we can assess the stability of the system:

$$\begin{aligned}\dot{\xi} &= \xi g'(S_1^{*(1)}) \\ &= \xi\Big(\frac{\mu_R\rho}{\delta_R+\eta S_1^{*(1)}} - \frac{\mu_R\rho\eta S_1^{*(1)}}{(\delta_R+\eta S_1^{*(1)})^2} - \frac{\gamma_1\mu_T}{\delta_T} - \delta_1\Big) \\ &= \xi\Big(\frac{\mu_R\rho}{\delta_R} - \frac{\gamma_1\mu_T}{\delta_T} - \delta_1\Big)\end{aligned}$$

the perturbation $\xi(t)$ will decay exponentially if $\mu_R\rho/\delta_R < \gamma_1\mu_T/\delta_T + \delta_1$ (that is, $S_1^{*(1)}$ is stable and $S_1^{*(2)}$ unstable under this condition) and will grow exponentially if $\mu_R\rho/\delta_R > \gamma_1\mu_T/\delta_T + \delta_1$ (that is, $S_1^{*(1)}$ is unstable and $S_1^{*(2)}$ stable under this condition). We observe the presence of a transcritical bifurcation as a function of the parameter μ_R (see Figure S1b)) with a critical value at:

$$\mu_R^c = \frac{\delta_1\delta_R}{\rho} + \frac{\delta_R\gamma_1\mu_T}{\rho\delta_T}$$

III. SURVIVAL OF SPECIES 2 (S_2) WITH TOXIC AND RESOURCES

The region of parameters where only S_2 survives is equivalent to this three dimensional system:

$$\dot{T} = \mu_T - \delta_T T \tag{9}$$

$$\dot{R} = \mu_R - \delta_R R - \eta S_1 R \tag{10}$$

$$\dot{S}_2 = \rho(1-\varepsilon)RS_2 - \gamma_2 TS_2 - \delta_2 S_2 \tag{11}$$

Assuming again that either T and R rapidly reach equilibrium due to their reaction time scale, we are left with the following 1D system:

$$\dot{S}_2 = \rho(1-\varepsilon)\Big(\frac{\mu_R}{\delta_R+\eta S_2}\Big)S_2 - \gamma_2\Big(\frac{\mu_T}{\delta_T+\varepsilon S_2}\Big)S_2 - \delta_2 S_2 = h(S_2) \tag{12}$$

which has the following fixed points:

$$S_2^{*(1)} = 0$$

$$\begin{aligned}S_2^{*(2,3)} = -\frac{1}{2\delta 2\varepsilon\eta}\Big[&\delta 2\delta_R\varepsilon + \delta 2\delta_T\eta + \gamma_2\mu_T\eta - \varepsilon\mu_R\rho + \varepsilon^2\mu_R\rho \pm \\ &\Big\{4\delta 2\varepsilon\eta\left(-\delta 2\delta_R\delta_T - \delta_R\gamma_2\mu_T + \delta_T\mu_R\rho - \delta_T\varepsilon\mu_R\rho\right) + \\ &\left[-\delta 2\delta_R\varepsilon - \delta 2\delta_T\eta - \gamma_2\mu_T\eta + \varepsilon\mu_R\rho - \varepsilon^2\mu_R\rho\right]^2\Big\}^{1/2}\Big]\end{aligned}$$

Using the linearization about $S_2^{*(1)}$ (see section I for a detailed derivation) we can assess the stability of the first fixed point:

$$\begin{aligned}\dot{\xi} &= \xi h'(S_2^{*(1)}) \\ &= \xi\Big(\frac{\rho(1-\varepsilon)\mu_R}{\delta_R+\eta S_2^{*(1)}} - \frac{\rho(1-\varepsilon)\mu_R\eta S_2^{*(1)}}{(\delta_R+\eta S_2^{*(1)})^2} - \frac{\gamma_2\mu_T}{\delta_T+\varepsilon S_2^{*(1)}} + \frac{\gamma_2\mu_T\varepsilon S_2^{*(1)}}{(\delta_T+\varepsilon S_2^{*(1)})^2} - \delta_2\Big) \\ &= \xi\Big(\frac{\rho(1-\varepsilon)\mu_R}{\delta_R} - \frac{\gamma_2\mu_T}{\delta_T} - \delta_2\Big)\end{aligned}$$

the perturbation $\xi(t)$ will decay exponentially if $\rho(1-\varepsilon)\mu_R/\delta_R < \gamma_2\mu_T/\delta_T + \delta_2$ (that is, $S_2^{*(1)}$ is stable under this condition) and will grow exponentially if $\rho(1-\varepsilon)\mu_R/\delta_R > \gamma_2\mu_T/\delta_T + \delta_2$ (that is, $S_2^{*(1)}$ is unstable under this

condition). We observe the presence of a transcritical bifurcation as a function of the parameter μ_R (see Figure S1 c)) with a critical value at:

$$\mu_R^c = \frac{\delta_2 \delta_R}{\rho(1-\varepsilon)} + \frac{\delta_R \gamma_2 \mu_T}{\rho(1-\varepsilon)\delta_T}$$

Notice that by the values of the critical μ_R we could already know the position of each critical value. The reproduction rate has a critical role in determining the existence of each species under this conditions, being S_1 more fit than S_2 despite the toxic degradation of the latter.

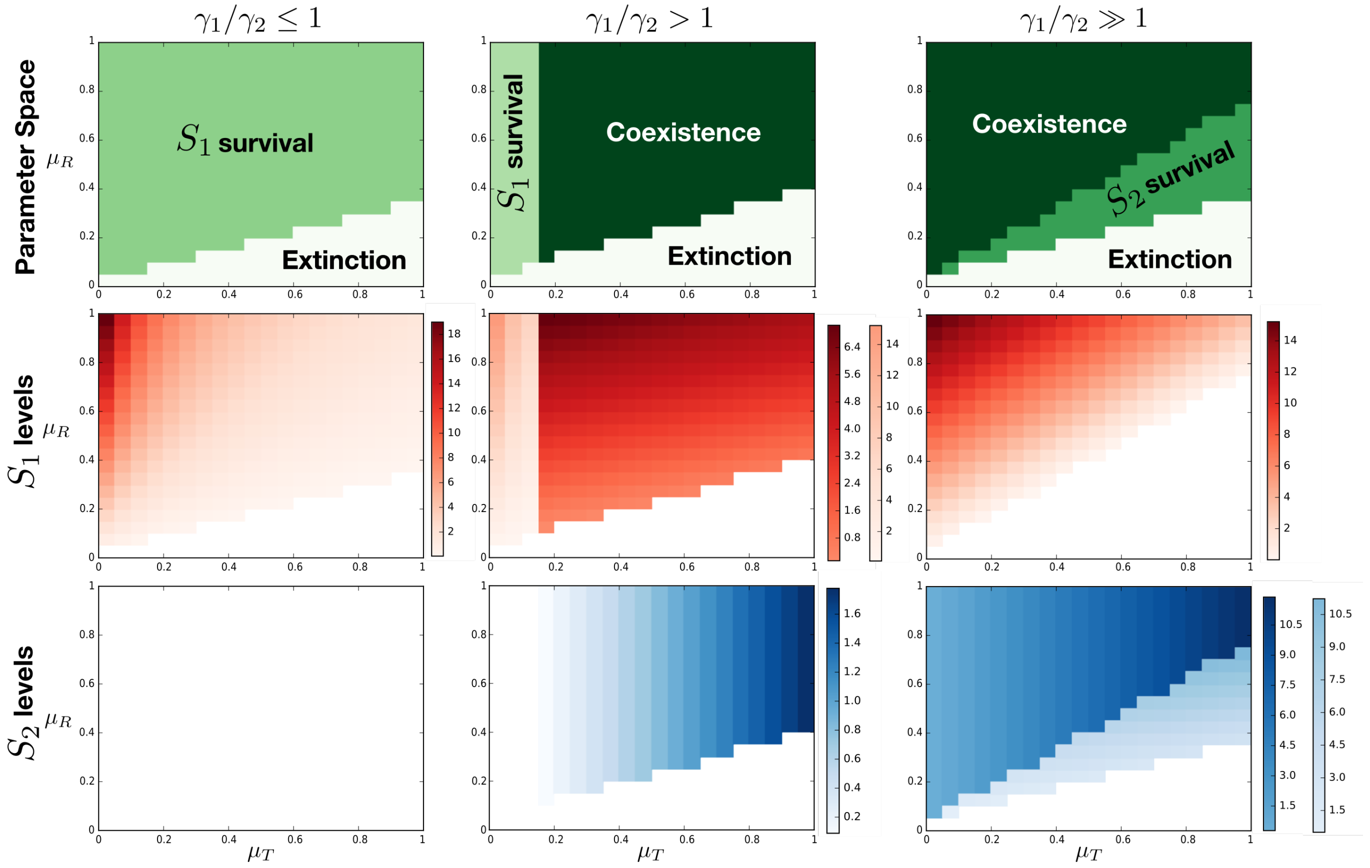

FIG. 2: Parameter space of the 2D system under different conditions of γ_1/γ_2 (each column) as a function of μ_T and μ_R For $\gamma_1/\gamma_2 \leq 1$, $\gamma_1 = \gamma_2 = 0.1$, for $\gamma_1/\gamma_2 > 1$, $\gamma_1 = 0.17$ and $\gamma_2 = 0.1$ and for $\gamma_1/\gamma_2 \gg 1$, $\gamma_1 = 0.3$ and $\gamma_2 = 0.1$

IV. TWO-DIMENSIONAL (S_1 AND S_2) RESOURCES AND TOXIC MODEL

Following the same strategy as in the previous sections, the four dimensional model presented in the paper can be reduced to two dimensions. This allows us to reach some interesting conclusions, as seen in the main text. Importantly, the main results of the 4D system still apply (compare Fig. 2 with Fig. 2 in main text). We are left with the following 2D system:

$$\dot{S}_1 = \rho \frac{\mu_R}{\delta_R + \eta(S_1 + S_2)} S_1 - \gamma_1 \frac{\mu_T}{\delta_T + \varepsilon S_2} S_1 - \delta_1 S_1 \tag{13}$$

$$\dot{S}_2 = \rho(1-\varepsilon) \frac{\mu_R}{\delta_R + \eta(S_1 + S_2)} S_2 - \gamma_2 \frac{\mu_T}{\delta_T + \varepsilon S_2} S_2 - \delta_2 S_2 \tag{14}$$

which has the same fixed points of the 4D system (see Section V), in this case just taking $FP_X = (S_1^*, S_2^*)$:

$$
\begin{aligned}
FP_1 &= (0,0) \\
FP_2 &= \left([\gamma_2 - \gamma_1(1-\varepsilon)] \left[\frac{\mu_R \rho}{\eta(\gamma_2\delta_1 - \gamma_1\delta_2)} - \frac{\mu_T}{\varepsilon[(1-\varepsilon)\delta_1 - \delta_2]} \right] - \frac{\delta_R}{\eta} + \frac{\delta_T}{\varepsilon}, \frac{\mu_T}{\varepsilon} \left[\frac{\gamma_2 - \gamma_1(1-\varepsilon)}{(1-\varepsilon)\delta_1 - \delta_2} \right] - \frac{\delta_T}{\varepsilon} \right) \\
FP_3 &= \left(\frac{\rho\mu_R\delta_T - \mu_T\gamma_1\delta_R - \delta_1\delta_T\delta_R}{\eta(\mu_T\gamma_1 + \delta_1\delta_T)}, 0 \right) \\
FP_{4,5} &= \Bigg(0, -\frac{1}{2\delta 2\varepsilon\eta} \Big[\delta 2\delta_R\varepsilon + \delta 2\delta_T\eta + \gamma_2\mu_T\eta - \varepsilon\mu_R\rho + \varepsilon^2\mu_R\rho \pm \\
&\quad \Big\{ 4\delta 2\varepsilon\eta \left(-\delta 2\delta_R\delta_T - \delta_R\gamma_2\mu_T + \delta_T\mu_R\rho - \delta_T\varepsilon\mu_R\rho \right) + \\
&\quad \left[-\delta 2\delta_R\varepsilon - \delta 2\delta_T\eta - \gamma_2\mu_T\eta + \varepsilon\mu_R\rho - \varepsilon^2\mu_R\rho \right]^2 \Big\}^{1/2} \Big] \Bigg)
\end{aligned}
$$

In order to perform the stability analysis of this system we again need to linearize it as it is a non-linear system. In order to do that, we define the perturbation around the fixed points of the system as: $u = S_1 - S_1^*$ and $v = S_2 - S_2^*$. To see whether the disturbance grows or decays, the derivation of differential equations for u and v is needed. Let's name the equations defining the system as follows: $\dot{S}_1 = f(S_1, S_2)$ and $\dot{S}_2 = g(S_1, S_2)$. Then, through the Taylor series expansion for a function of two variables and ignoring higher order terms, we obtain:

$$
\begin{aligned}
\dot{u} &= u\frac{\partial f}{\partial S_1} + v\frac{\partial f}{\partial S_2} \\
\dot{v} &= u\frac{\partial g}{\partial S_1} + v\frac{\partial g}{\partial S_2}
\end{aligned}
$$

where all the partial derivatives are to be evaluated at (S_1^*, S_2^*). Hence, the disturbance (u, v) evolves according to:

$$
\begin{pmatrix} \dot{u} \\ \\ \dot{v} \end{pmatrix} = \begin{pmatrix} \dfrac{\partial f}{\partial S_1} & \dfrac{\partial f}{\partial S_2} \\ \dfrac{\partial g}{\partial S_1} & \dfrac{\partial g}{\partial S_2} \end{pmatrix}_{(S_1^*, S_2^*)} \begin{pmatrix} u \\ \\ v \end{pmatrix}
$$

which is the linearized system, that we will express as $\dot{\mathbf{u}} = A\mathbf{u}$, with

$$
A = \begin{pmatrix} \frac{\partial f}{\partial S_1} & \frac{\partial f}{\partial S_2} \\ \frac{\partial g}{\partial S_1} & \frac{\partial g}{\partial S_2} \end{pmatrix}_{(S_1^*, S_2^*)}
$$

being the Jacobian matrix of the system at the fixed point (S_1, S_2). Then we will proceed with the stability analysis typical of linear systems to assess the stability of our linearized system about (S_1^*, S_2^*). Restricting to straight-line solutions and assuming that the solutions of the linearized system are to be of the form $\mathbf{u}(t) = \mathbf{w}e^{\lambda t}$, being $\mathbf{w}$ a constant vector referring to the direction of the solution in the plane, we proceed as follows: $\dot{\mathbf{u}} = \mathbf{w}\lambda e^{\lambda t} = A\mathbf{u} = A(\mathbf{w}e^{\lambda t}) = e^{\lambda t}A\mathbf{w}$. Such solutions exist if we can find a $\mathbf{w}$ and a λ such that $A\mathbf{w} = \lambda\mathbf{v}$. Using linear algebra, we know that λ is given by $det(A - \lambda I) = 0$, which gives the so-called characteristic equation.

The jacobian matrix $\mathcal{J}$ reads

$$
\mathcal{J} = \begin{pmatrix} \frac{\mu_R\rho}{\delta_R + \eta(S_1+S_2)} - \frac{\mu_R\rho\eta S_1}{(\delta_R+\eta(S_1+S_2))^2} - \frac{\gamma_1\mu_T}{\delta_T + \varepsilon S_2} - \delta_1 & \frac{-\mu_R\rho\eta S_1}{(\delta_R+\eta(S_1+S_2))^2} + \frac{\gamma_1\mu_T\varepsilon S_1}{(\delta_T+\varepsilon S_2)^2} \\ -\frac{\rho(1-\varepsilon)\mu_R\eta S_2}{(\delta_R+\eta(S_1+S_2))^2} & \frac{\rho(1-\varepsilon)\mu_R}{\delta_R+\eta(S_1+S_2)} - \frac{\rho(1-\varepsilon\mu_R\eta S_2)}{(\delta_R+\eta(S_1+S_2))^2} - \frac{\gamma_2\mu_T}{\delta_T+\varepsilon S_2} + \frac{\gamma_2\mu_T\varepsilon S_2}{(\delta_T+\varepsilon S_2)^2} - \delta_2 \end{pmatrix}_{(S_1^*, S_2^*)}
$$

substituting $(S_1^*, S_2^*) = (0, 0)$,

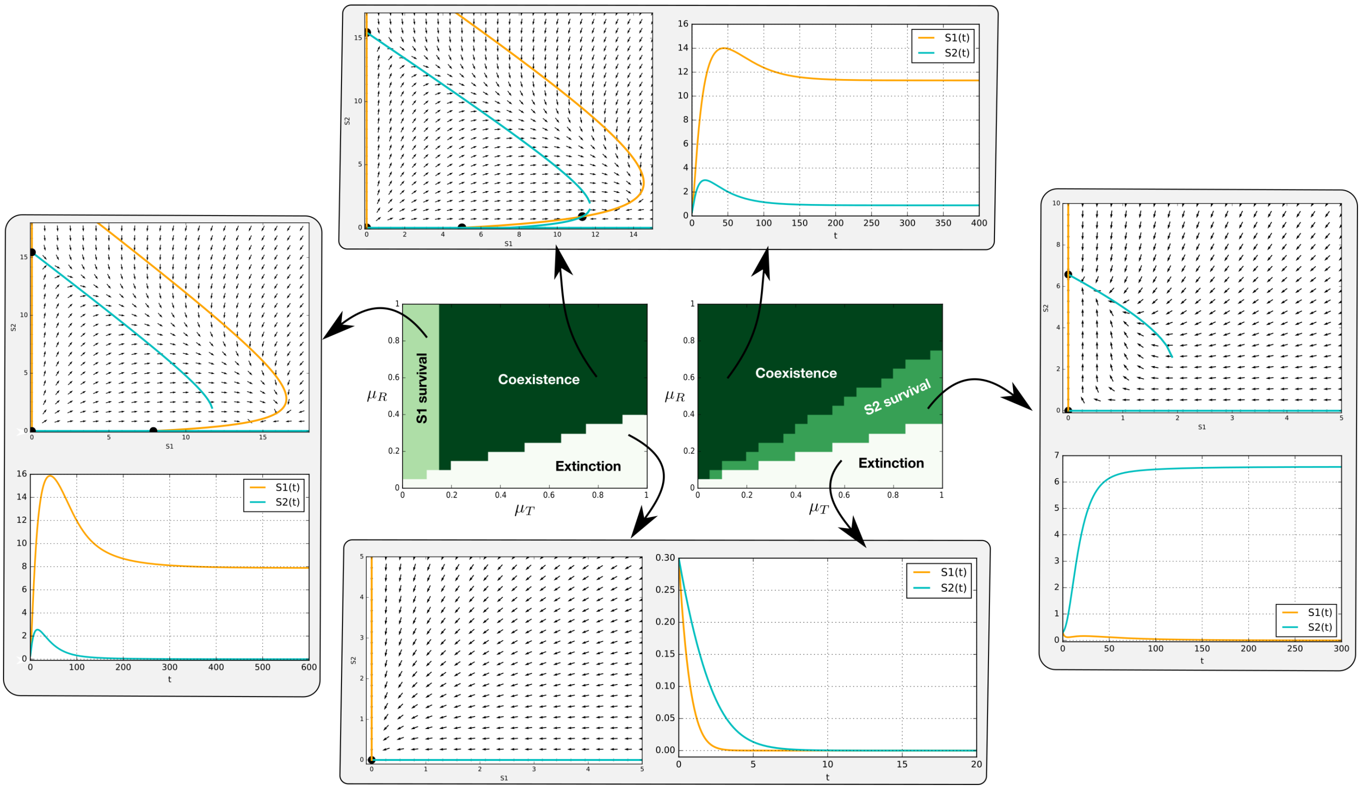

FIG. 3: Phase portrait with nullclines and trajectories of the four stability situations (coexistence of S_1 and S_2, survival of S_1 or S_2 and extinction of both) for the 2D version of the model.

$$\mathcal{J} = \begin{pmatrix} \dfrac{\mu_R \rho}{\delta_R} - \dfrac{\gamma_1 \mu_T}{\delta_T} - \delta_1 & 0 \\ 0 & \dfrac{\rho(1-\varepsilon)\mu_R}{\delta_R} - \dfrac{\gamma_2 \mu_T}{\delta_T} - \delta_2 \end{pmatrix}$$

We obtain the eigenvalues directly from the expression $det(A - \lambda I) = 0$, without the need to explicit the characteristic equation

$$det \begin{pmatrix} \dfrac{\mu_R \rho}{\delta_R} - \dfrac{\gamma_1 \mu_T}{\delta_T} - \delta_1 - \lambda & 0 \\ 0 & \dfrac{\rho(1-\varepsilon)\mu_R}{\delta_R} - \dfrac{\gamma_2 \mu_T}{\delta_T} - \delta_2 - \lambda \end{pmatrix} = 0$$

The eigenvalues read

$$\lambda_1 = \frac{\mu_R \rho}{\delta_R} - \frac{\gamma_1 \mu_T}{\delta_T} - \delta_1$$
$$\lambda_2 = \frac{\rho(1-\varepsilon\mu_R)}{\delta_R} - \frac{\gamma_2 \mu_T}{\delta_T} - \delta_2$$

and their signs will indicate the stability of the fixed point around which we have linearized the system $(S_1^*, S_2^*) =$

$(0,0)$:

$$\begin{aligned}
&\text{if } \lambda_1 < 0 \text{ and } \lambda_2 < 0, (S_1^*, S_2^*) = (0,0) \text{ is STABLE}\\
&\text{if } \lambda_1 < 0 \text{ and } \lambda_2 > 0, (S_1^*, S_2^*) = (0,0) \text{ is a SADDLE NODE}\\
&\text{if } \lambda_1 > 0 \text{ and } \lambda_2 < 0, (S_1^*, S_2^*) = (0,0) \text{ is SADDLE NODE}\\
&\text{if } \lambda_1 > 0 \text{ and } \lambda_2 > 0, (S_1^*, S_2^*) = (0,0) \text{ is UNSTABLE}
\end{aligned}$$

The rest of the the Jacobian matrices (with the other fixed points substituted) are too involved and are not presented.

A. Phase portrait, nullclines and trajectories

The nullclines of the 2D system are obtained through the isolation of S_1 and S_2 in their respective differential equations, meaning that along that line/curve, $\dot{S_1}$ and $\dot{S_2}$ do not change:

$$\begin{aligned}
\dot{S_1} &= \rho \frac{\mu_R}{\delta_R + \eta(S_1 + S_2)} S_1 - \gamma_1 \frac{\mu_T}{\delta_T + \varepsilon S_2} S_1 - \delta_1 S_1 = 0\\
\dot{S_2} &= \rho(1-\varepsilon) \frac{\mu_R}{\delta_R + \eta(S_1 + S_2)} S_2 - \gamma_2 \frac{\mu_T}{\delta_T + \varepsilon S_2} S_2 - \delta_2 S_2 = 0
\end{aligned}$$

We are left with the following expression for the nullclines ($n_1 ... n_5$):

$$\begin{aligned}
n_1 &\to S_1 = 0\\
n_2 &\to S_1 = \frac{-\delta_1\delta_R\delta_T - \delta_R\gamma_1\mu_T + \delta_T\mu_R\rho - \delta_1\delta_R\varepsilon S_2 - \delta_1\delta_T\eta S_2 - \eta\gamma_1\mu_T S_2 + \varepsilon\mu_R\rho S_2 - \delta_1\varepsilon\eta S_2^2}{\eta(\delta_1\delta_T + \gamma_1\mu_T + \delta_1\varepsilon S_2)}\\
n_3 &\to S_2 = 0\\
n_{4,5} &\to S_2 = -\frac{1}{2\delta_2\varepsilon\eta}\Big(\delta 2\delta_R\varepsilon + \delta_2\delta_T\eta + \eta\gamma_2\mu_T - \varepsilon\mu_R\rho + \varepsilon^2\mu_R\rho + \delta_2\varepsilon\eta S_1 \pm\\
&\Big\{(-\delta_2\delta_R\varepsilon - \delta_2\delta_T\eta - \eta\gamma_2\mu_T + \varepsilon\mu_R\rho - \varepsilon^2\mu_R\rho - \delta_2\varepsilon\eta S_1)^2 +\\
&4\delta_2\varepsilon\eta(-\delta_2\delta_R\delta_T - \delta_R\gamma_2\mu_T + \delta_T\mu_R\rho - \delta_T\varepsilon\mu_R\rho - \delta_2\delta_T\eta S_1 - \eta\gamma_2\mu_T S_1)\Big\}^{1/2}\Big)
\end{aligned}$$

In Fig. 3 the nullclines relevant for the fixed points represented are plotted, together with the vector field to see the trajectory followed by the system given a set of parameters. The fixed points which are physical for that given set of parameters are also plotted in the phase portrait. The trajectories correspondent to the phase portraits are also shown. We give en example of each of the equilibrium points of the system (extinction, S_1 survival, S_2 survival and coexistence).

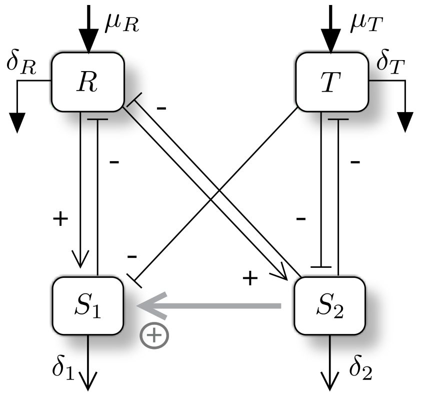

FIG. 4: 4D modeled system.

V. LINEAR STABILITY ANALYSIS OF THE 4D SYSTEM

The fixed points of the system presented in the main text (and depicted in Fig. **??**)read

$$
\begin{aligned}
\mathbf{P_1} &= \Big(\frac{\mu_T}{\delta_T}, \frac{\mu_R}{\delta_R}, 0, 0\Big) \\
\mathbf{P_2} &= \Bigg(\frac{(1-\varepsilon)\delta_1 - \delta_2}{\gamma_2 - \gamma_1(1-\varepsilon)}, \frac{\gamma_2\delta_1 - \gamma_1\delta_2}{\rho(\gamma_2 - \gamma_1(1-\varepsilon))}, \\
&\quad [\gamma_2 - \gamma_1(1-\varepsilon)]\left[\frac{\mu_R\rho}{\eta(\gamma_2\delta_1 - \gamma_1\delta_2)} - \frac{\mu_T}{\varepsilon[(1-\varepsilon)\delta_1 - \delta_2]}\right] \\
&\quad - \frac{\delta_R}{\eta} + \frac{\delta_T}{\varepsilon}, \frac{\mu_T}{\varepsilon}\left[\frac{\gamma_2 - \gamma_1(1-\varepsilon)}{(1-\varepsilon)\delta_1 - \delta_2}\right] - \frac{\delta_T}{\varepsilon}\Bigg) \\
\mathbf{P_3} &= \left(\frac{\mu_T}{\delta_T}, \frac{\mu_T\gamma_1 + \delta_1\delta_T}{\rho\delta_T}, \frac{\rho\mu_R\delta_T - \mu_T\gamma_1\delta_R - \delta_1\delta_T\delta_R}{\eta(\mu_T\gamma_1 + \delta_1\delta_T)}, 0\right) \\
\mathbf{P_{4,5}} &= \left(\frac{\mu_T}{\delta_T + \varepsilon S_2^*}, \frac{\mu_R}{\delta_T + \eta S_2^*}, 0, S_2^*\right)
\end{aligned}
$$

with

$$
\begin{aligned}
S_2^* = -\frac{1}{2\delta_2\varepsilon\eta}\Big[&\delta_2\delta_R\varepsilon + \delta_2\delta_T\eta + \gamma_2\mu_T\eta - \varepsilon\mu_R\rho + \varepsilon^2\mu_R\rho \pm \\
&\Big\{4\delta_2\varepsilon\eta\left(-\delta_2\delta_R\delta_T - \delta_R\gamma_2\mu_T + \delta_T\mu_R\rho - \delta_T\varepsilon\mu_R\rho\right) + \\
&\left[-\delta_2\delta_R\varepsilon - \delta_2\delta_T\eta - \gamma_2\mu_T\eta + \varepsilon\mu_R\rho - \varepsilon^2\mu_R\rho\right]^2\Big\}^{1/2}\Big]
\end{aligned}
$$

The detailed procedure to assess the stability of the fixed points of a non-linear system can be found at the section **S-VI: Two-Dimensional (S_1 and S_2) resources and toxic model**. The Jacobian matrix $\mathcal{J}$ of the 4D system reads,

$$
\mathcal{J} = \begin{pmatrix}
-\delta_T - \varepsilon S_2 & 0 & 0 & -\varepsilon T \\
0 & -\delta_R - \eta(S_1 + S_2) & -\eta R & -\eta R \\
-\gamma_1 S_1 & \rho S_1 & \rho R - \gamma_1 T - \delta_1 & 0 \\
-\gamma_2 S_2 & \rho(1-\varepsilon)S_2 & 0 & \rho(1-\varepsilon)R - \gamma_2 T - \delta_2
\end{pmatrix}_{(T^*, R^*, S_1^*, S_2^*)}
$$

Substituting $\mathbf{P_1}$,

$$
\mathcal{J} = \begin{pmatrix}
-\delta_T & 0 & 0 & -\dfrac{\varepsilon\mu_T}{\delta_T} \\
0 & -\delta_R & -\dfrac{\eta\mu_R}{\delta_R} & -\dfrac{\eta\mu_R}{\delta_R} \\
0 & 0 & \dfrac{\rho\mu_R}{\delta_R} - \dfrac{\gamma_1\mu_T}{\delta_T} - \delta_1 & 0 \\
0 & 0 & 0 & \dfrac{\rho(1-\varepsilon)\mu_R}{\delta_R} - \dfrac{\gamma_2\mu_T}{\delta_T} - \delta_2
\end{pmatrix}
$$

Applying $det(A - \lambda I) = 0$, we can directly obtain the eigenvalues, which read

$$
\begin{aligned}
\lambda_1 &= -\delta_T \\
\lambda_2 &= -\delta_R \\
\lambda_3 &= \frac{\rho\mu_R}{\delta_R} - \frac{\gamma_1\mu_T}{\delta_T} - \delta_1 \\
\lambda_4 &= \frac{\rho(1-\varepsilon)\mu_R}{\delta_R} - \frac{\gamma_2\mu_T}{\delta_T} - \delta_2
\end{aligned}
$$

From which we only can infer that,

$$\text{if } \frac{\rho\mu_R}{\delta_R} < \frac{\gamma_1\mu_T}{\delta_T} + \delta_1 \text{ AND if } \frac{\rho(1-\varepsilon)\mu_R}{\delta_R} < \frac{\gamma_2\mu_T}{\delta_T} + \delta_2$$

then, $\mathbf{P_1}$ is stable, as λ_1 and λ_2 will always be negative, as the constants are by definition positive values, and λ_3 and λ_4 take also negative values if the conditions just stated are accomplished.

The substitution of the other fixed points into the Jacobian matrix $\mathcal{J}$ leads to complicated expressions, from which the eigenvalues obtained are too involved (contain tens or hundreds of terms) in order to be presented in this work (the usage of the software Wolfram Mathematica was used to obtain it).

A. Conditions for extinction: counterpart to numerical simulations

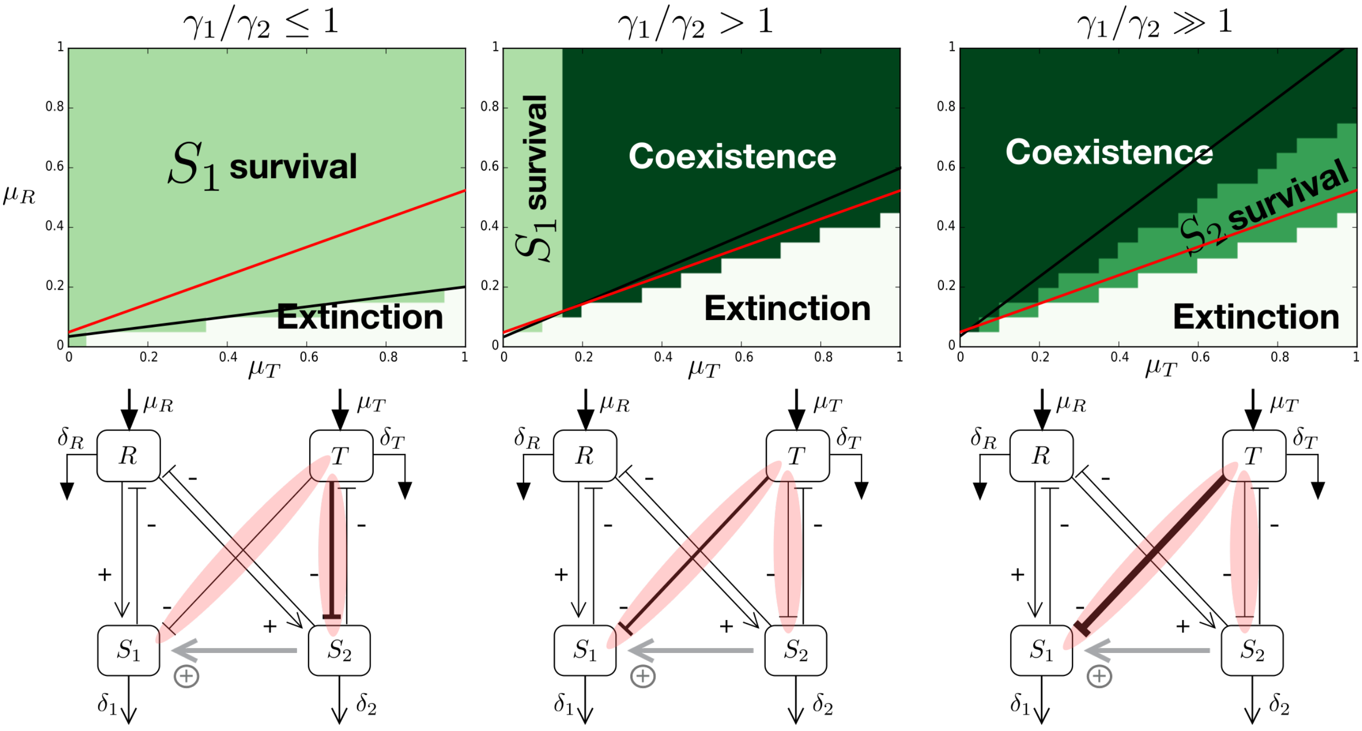

FIG. 5: Conditions for extinction in the 4D version of the model using the eigenvalues obtained from the Jacobian matrix ($\mathcal{J}|_{\mathbf{P_1}}$) (extinction fixed point).

As we have previously observed, the balance between μ_R and μ_T determines the limits of the system existence, that is, the limit between extinction and survival of one of the species or both. This can be confirmed analytically using the eigenvalues obtained from the Jacobian matrix ($\mathcal{J}|_{\mathbf{P_1}}$) (extinction fixed point). From section S1-SM, we know that λ_1 and λ_2 are always negative. The values of λ_3 and λ_4 can be written as a function of μ_R and μ_T, which, together, determine the limits between extinction and system's viability:

$$\lambda_3 = \mathcal{X}\mu_R - \mathcal{Y}\mu_T - \mathcal{Z}$$
$$\lambda_4 = \mathcal{M}\mu_R - \mathcal{N}\mu_T - \mathcal{O}$$

with $\mathcal{X} = \frac{\rho}{\delta_R}$, $\mathcal{Y} = \frac{\gamma_1}{\delta_T}$ and $\mathcal{Z} = \delta_1$, for λ_3 and $\mathcal{M} = \frac{\rho(1-\varepsilon)}{\delta_R}$, $\mathcal{N} = \frac{\gamma_2}{\delta_T}$ and $\mathcal{L} = \delta_2$, for λ_4. As we need both λ_3 and λ_4 to be negative so that the Extinction fixed point ($\mathbf{P_1}$) is stable, the above expressions can be set as inequalities as follows,

$$\lambda_3 \Longrightarrow \mu_R < \frac{\mathcal{Y}\mu_T + \mathcal{Z}}{\mathcal{X}} \tag{15}$$

$$\lambda_4 \Longrightarrow \mu_R < \frac{\mathcal{N}\mu_T + \mathcal{O}}{\mathcal{M}} \tag{16}$$

so that if we plot $\mu_R = f(\mu_T)$ for both eigenvalues in the same 2D plot, the region where both inequality conditions hold will fulfill the condition of stability of the extinction fixed point $\mathbf{P_1}$. In Fig. S4 we can observe how the Parameter Spaces obtained numerically fulfill the condition just mentioned when the two functions are plotted above them.

On the other hand, the values of ε and γ_1, the ones confirming the positive correlation of the links conforming the cooperative interaction from S_2 to S_1, have also a critical role either in allowing the coexistence of the species and also, again, defining a limit between extinction or absence of it. This can also be confirmed analytically following the same strategy as in the case of μ_R and μ_T. The eigenvalues λ_3 and λ_4 depend on γ_1 and ε, respectively,

$$\lambda_3 = \mathcal{H} - \mathcal{I}\gamma_1$$
$$\lambda_4 = -\mathcal{K}\varepsilon + \mathcal{L}$$

with $\mathcal{H} = \frac{\rho\mu_R}{\delta_R} - \delta_1$ (which is assumed to be positive) and $\mathcal{I} = \mu_T/\delta_T$, for λ_3, and $\mathcal{K} = \frac{\rho\mu_R}{\delta_R}$ and $\mathcal{L} = \frac{\rho\mu_R}{\delta_R} - \gamma_2\mu_T/\delta_T - \delta_2$ (also assumed to be positive), for λ_4. Therefore, we can again write two inequalities defining again the limit of $\mathbf{P_1}$ stability,

$$\lambda_3 \Longrightarrow \gamma_1 > \frac{\mathcal{H}}{\mathcal{I}} \tag{17}$$

$$\lambda_4 \Longrightarrow \varepsilon > \frac{\mathcal{L}}{\mathcal{K}} \tag{18}$$

so that when γ_1 and ε have values above each particular scalar, $\mathbf{P_1}$ is stable. For the set of values used to build the $\varepsilon - \gamma_1$ parameter space of Fig. 3 in the main text, the inequalities that hold are: $\varepsilon > 0.6$ and $\gamma_1 > 0.28$ (check Fig. S5a). As we can observe in Fig. 3 of the main text, there is a region where $\mathbf{P_1}$ is stable where we find coexistence of the species. This is explained by the fact that in this region of the parameter space a bistability behaviour is found, with both $\mathbf{P_1}$ and $\mathbf{P_2}$ stable. In Fig. S5 we observe the bistability behaviour in the 2D model counterpart (which it has been shown to display the same properties than the 4D model). A progressive zoom in is shown in Fig. S5 from left to right to correctly appreciate the bistability behaviour described.

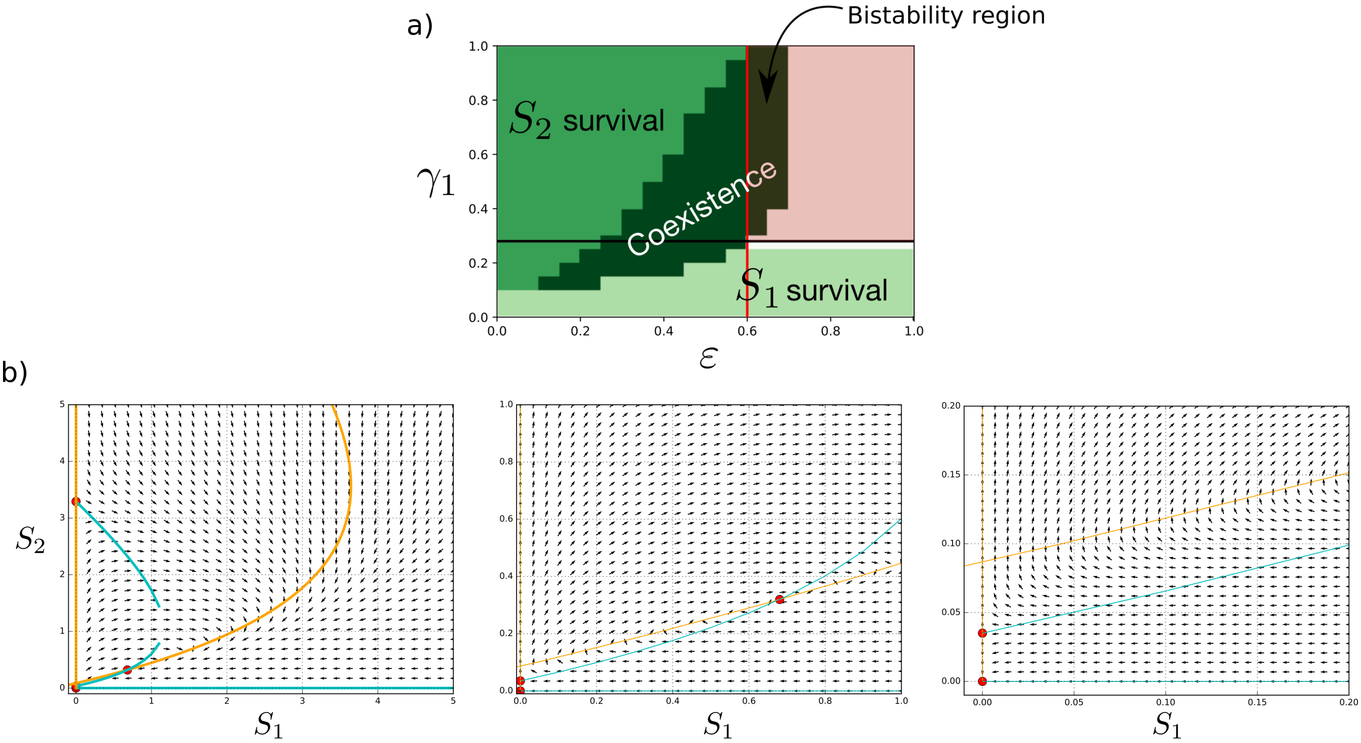

FIG. 6: **Bistability in a region of the parameter space ε vs. γ_1.** a) Parameter space ε vs. γ_1 with the lines showing the inequalities 17 and 18 and the region delimiting stability of the extinction fixed point (blurred red). b) Bistability behaviour checked in the 2D version of the model presented in the main text. Progressive zoom in is found from left to right to correctly appreciate the bistability behaviour of the extinction and coexistence fixed points.

VI. QUALITATIVE STABILITY ANALYSIS OF THE 4D SYSTEM

Qualitative stability is a quite useful method to assess the stability of a multispecies system of interactions either when no quantitative information about the system is available or when assessing the stability analytically is intractable or extremely involved. [1–5]. It can also be considered a shortcut method for investigating large systems that needs little if any computation[1]. Numerical approximations always imply assumptions about the magnitude's values, while qualitative approaches can result in valuable conclusions regarding the stability of your system only knowing the topological structure of the trophic web, i.e., knowing only the signs (+, -, or 0) of the interactions between the various species[4]. On the other hand, it could be discussed that the partial specification of a system may sometimes be closer to the biological reality than a complete one[2], and therein the importance of qualitative stability. Motivation for this method actually came from economics by Quirk and Ruppert[6]. Later on, the method was further developed and applied to the ecology field by May[4, 5], Jeffries[7] and Levins[2, 3][1].

$$\mathbf{A} = \begin{pmatrix} -a_{TT} & 0 & 0 & -a_{TS_2} \\ 0 & -a_{RR} & -a_{RS_1} & -a_{RS_2} \\ -a_{S_1T} & a_{S_1R} & 0 & 0 \\ -a_{S_2T} & a_{S_2R} & 0 & 0 \end{pmatrix}$$

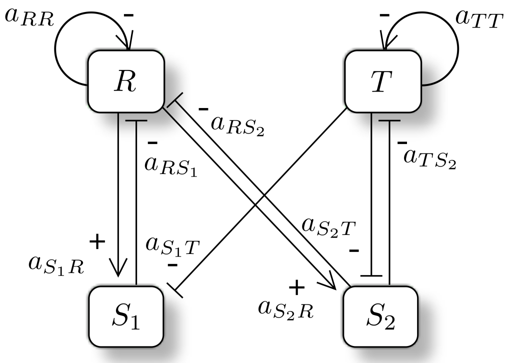

FIG. 7: Digraph from community matrix.

First I will present the qualitative stability analysis rooted in economics applied to our system and then the Loop Analysis method, considered also to be a qualitative method but in this case rooted in Linear Stability Analysis and Routh-Hurwitz criteria. Using the latter method we are able to set out some conditions that must hold so that our system is stable and, besides, we are able to crosscheck these results with our numeric simulations.

A. Qualitative Stability conditions rooted in economics

Following Quirk and Ruppert (1965)[6], May (1973)[4, 5] outlined five conditions for qualitative stability. Suppose a_{ij} is the ijth element of the matrix of signs $\mathbf{A}$.

$$\mathbf{A} = \begin{pmatrix} a_{TT} & a_{TR} & a_{TS_1} & a_{TS_2} \\ a_{RT} & a_{RR} & a_{RS_1} & a_{RS_2} \\ a_{S_1T} & a_{S_1R} & a_{S_1S_1} & a_{S_1S_2} \\ a_{S_2T} & a_{S_2R} & a_{S_1S_2} & a_{S_2S_2} \end{pmatrix}$$

The matrix of signs just contains the signs of the elements of the so-called Jacobian matrix or community matrix, which is obtained through the linearization of the set of differential equations describing the rate of change of the nth variables of our system.The elements a_{ij} of the community matrix are readily interpreted as the effect of X_j on the level of X_i. Our system's community matrix represented just with a_{ij} entries is shown in Fig. 6 together with the digraph showing the signs interactions between the elements of the system. The actual Jacobian matrix of our system was already calculated in section **S-I**.

In order to check if the system is qualitatively stable (which will mean it is stable in the ordinary sense -but the converse is not true)[1], it is necessary for all of the following conditions to hold:

1. $a_{ii} \leq 0$ for all i.
2. $a_{ii} < 0$ for at least one i.
3. $a_{ij}a_{ji} \leq 0$ for all $i \neq j$.

4. $a_{ij}a_{jk}...a_{qr}a_{ri} = 0$ for any sequences of three or more distinct indices $i, j, k, ..., q, r$.
5. det $\mathbf{A} \neq 0$.

These conditions are biologically interpreted as follows[1]

1. No species exerts positive feedback on itself.
2. At least one species is self-regulating.
3. The members of any given pair of interacting species must have opposite effects on each other.
4. There are no closed chains of interactions among three or more species.
5. There is no species that is unaffected by interactions with itself or with other species, that is, no node is devoid of input arrows.

Our system fulfills conditions **1** (no positive feedback exists), **2** (R and T are self-regulating) and **5** (all nodes have at least one input arrow), but breaks condition **3** (due to the pair of interactions involving T and S_2) and **4** (there is a four species cycle -$a_{S_2R}a_{RS_1}a_{S_1T}a_{TS_2}$). Therefore, the system cannot be said to be qualitatively stable, but it does not imply it can be stable under appropriate magnitude values of the interactions. In other words, our system can still be *conditionally stable.*

As this method is not universally applicable and the understanding of its proofs requires a deep knowledge of matrices beyond linear algebra, it is viewed as secondary[1]. Moreover, most natural webs will easily violate both 3 and 4 conditions, which makes the method not so general. However, the fact that commensal, amensal and predator-prey interactions are consistent qualitative interactions, while symbiotic and competitive ones are not, can be a feature to be taken into account in the open question of the relation between complexity and stability[4].

B. The Loop Analysis Method

The mathematical methods used in the method of Loop Analysis depend on the equivalence between differential equations near equilibrium, on the one hand, and matrices and their diagrams, on the other[3]. In order to fully understand the method presented here we refer the reader to Levins 1974 and 1975 [2, 3]. Here we only present the basic concepts needed to develop the analysis. If the reader is interested in this method we refer him/her to a paper where another alternative method also based on Routh-Hurwitz criteria is developed also for qualitative stability purposes[8].

As in the previous section, we first need to obtain the community matrix of our set of equations (see Figure 6). In this digraph, we observe a set of loops: self-loops of length one (a_{RR} and a_{TT}), loops of length two ($a_{RS_1}a_{S_1R}$, $a_{RS_2}a_{S_2R}$ and $a_{TS_2}a_{S_2T}$), no loops of length three and a single loop of length four ($a_{S_2R}a_{RS_1}a_{S_1T}a_{TS_2}$). Two loops not sharing vertices in common are called *disjunct* loops, while loops sharing a vertex are called *conjunct.* Notice also the self-damping of R and T, which does not exist in species S_1 and S_2. This is due to the fact that the growth of the species S_1 and S_2 is dependent on R, so in these cases neither self-damping nor self-acceleration is considered[3]. As in the previous method, just the signs of the entries of this matrix are needed to proceed with the loop analysis.

As we have previously advanced in the section's introduction, this method is rooted in Linear Stability Analysis and Routh-Hurwitz criteria. The most important ingredient of this method is to assess that the determinant of a matrix is equivalent to the sum of products of loops as follows:

$$D_n = \sum (-1)^{n-m} L(m, n) \tag{19}$$

where $L(m, n)$ is the product of n links (i.e. coefficients a_{ij}) which form m disjunct loops. This interpretation was first presented by Mason [9] and S. Wright [10] but it was not until Levins 1974 [2] that this interpretation gained significance for qualitative analysis purposes[3]. Following this objective, it is useful to introduce an expression which defines the *feedback* at level k in a system:

$$F_k = \sum (-1)^{m+1} L(m, k) \tag{20}$$

being F_k the sum of all possible products that involve just k vertices or variables and consist of, importantly, *disjunct* loops. The sign is adjusted so that if all loops are negative so it is F_k[3].

The conditions for a system to be stable just having the signs of the feedback loops F_k's are the following:

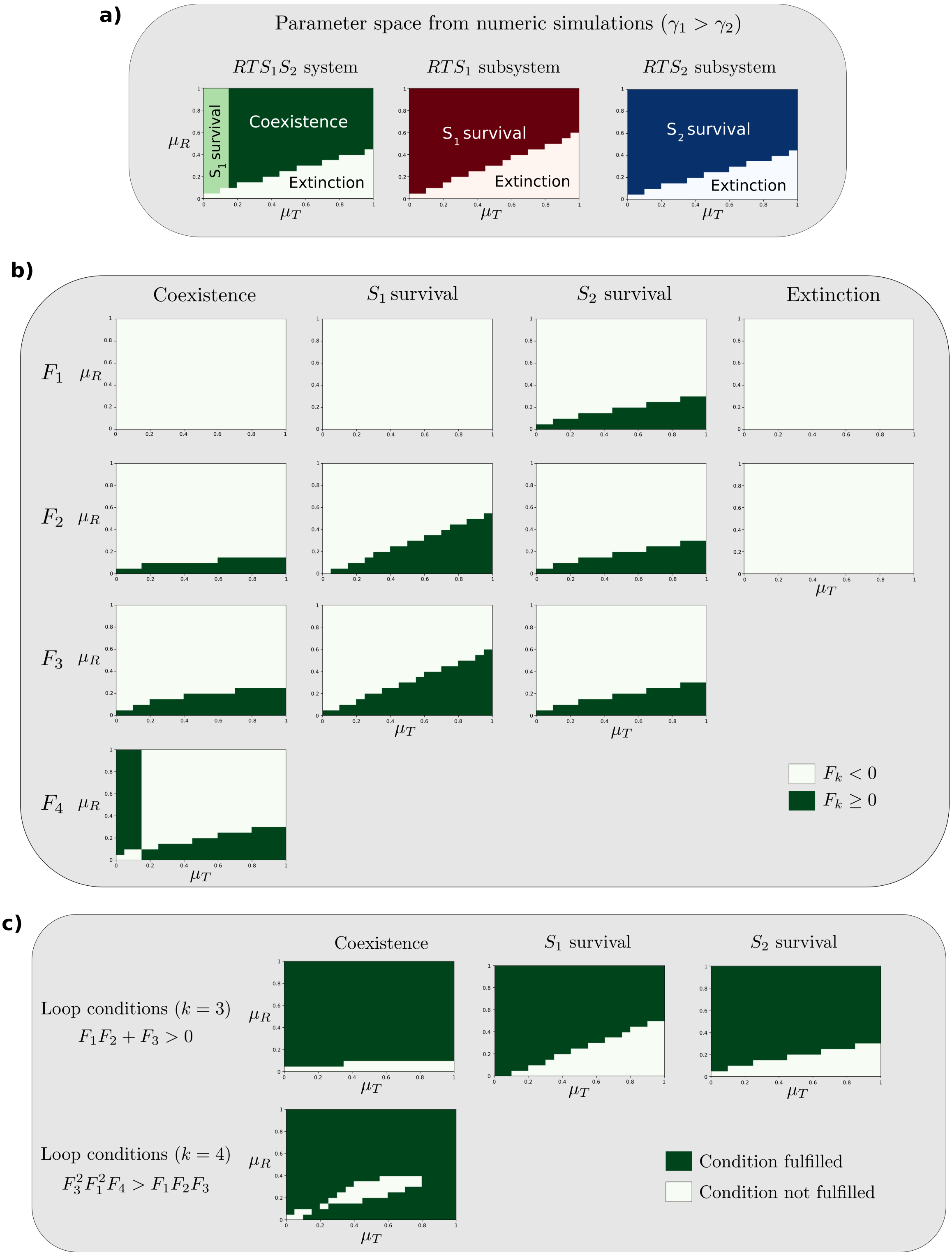

FIG. 8: Parameter space μ_T vs μ_R (condition $\gamma_1/\gamma_2 > 1$). Crosscheck between numerical results and qualitative stability analysis using the Loop Analysis method. **a)** Parameter space obtained from numerical simulations. Three initial conditions are shown, from left to right: both species present at $t = 0$, only S_1 present at $t = 0$ and only S_2 present at $t = 0$. **b)** Checking the sign of each feedback at level k (F_k's, from $k = 1...4$), to check if condition 1 section VI-B (see text) is fulfilled. **c)** Checking if the loop overwhelming conditions are fulfilled (see condition 2 section VI-B in text).

1. All loops must be **negative**.

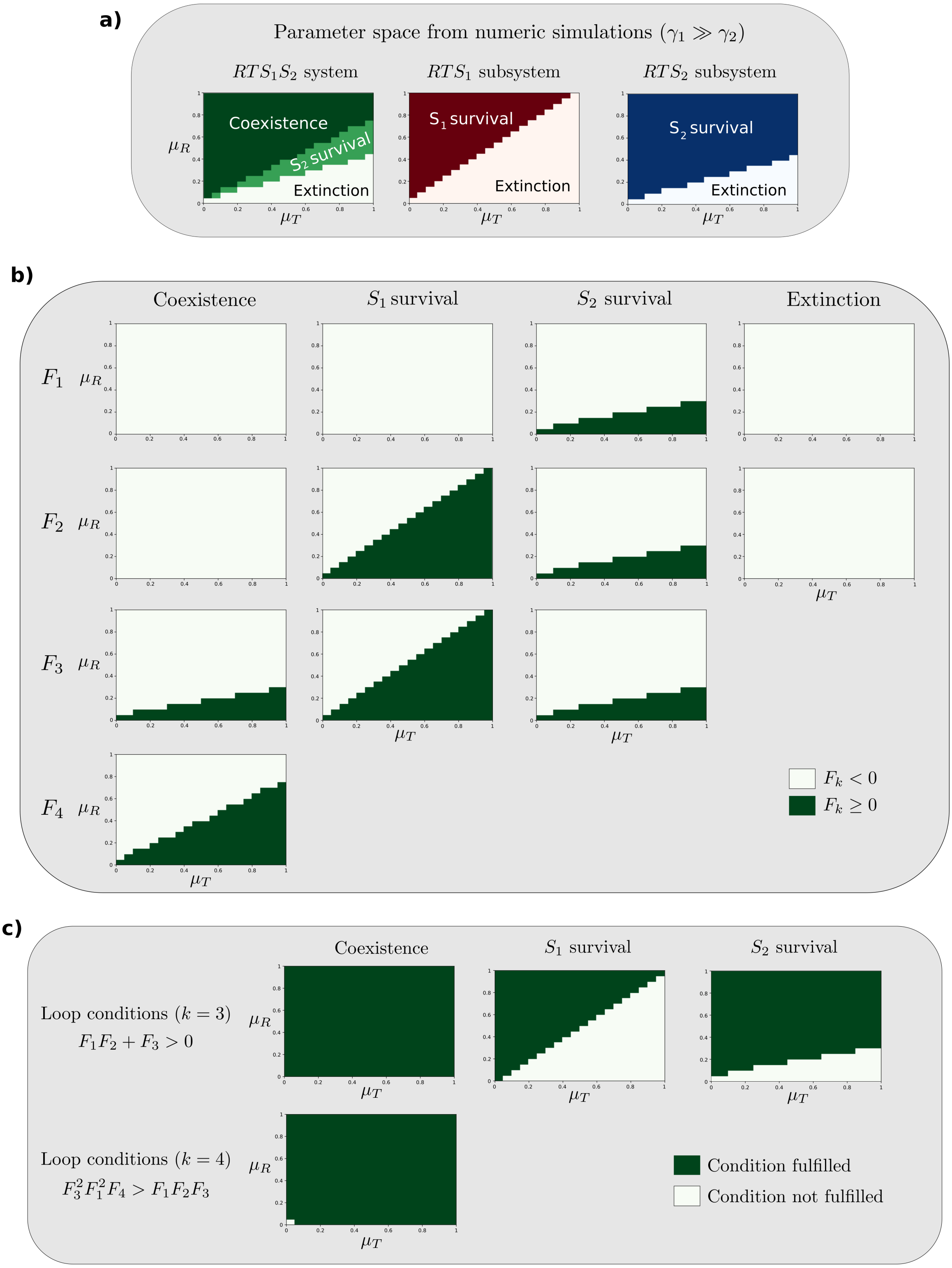

FIG. 9: Parameter space μ_T vs μ_R (condition $\gamma_1/\gamma_2 \gg 1$). Crosscheck between numerical results and qualitative stability analysis using the Loop Analysis method. **a)** Parameter space obtained from numerical simulations. Three initial conditions are shown, from left to right: both species present at $t = 0$, only S_1 present at $t = 0$ and only S_2 present at $t = 0$. **b)** Checking the sign of each feedback at level k (F_k's, from $k = 1...4$), to check if condition 1 section VI-B (see text) is fulfilled. **c)** Checking if the loop overwhelming conditions are fulfilled (see condition 2 section VI-B in text).

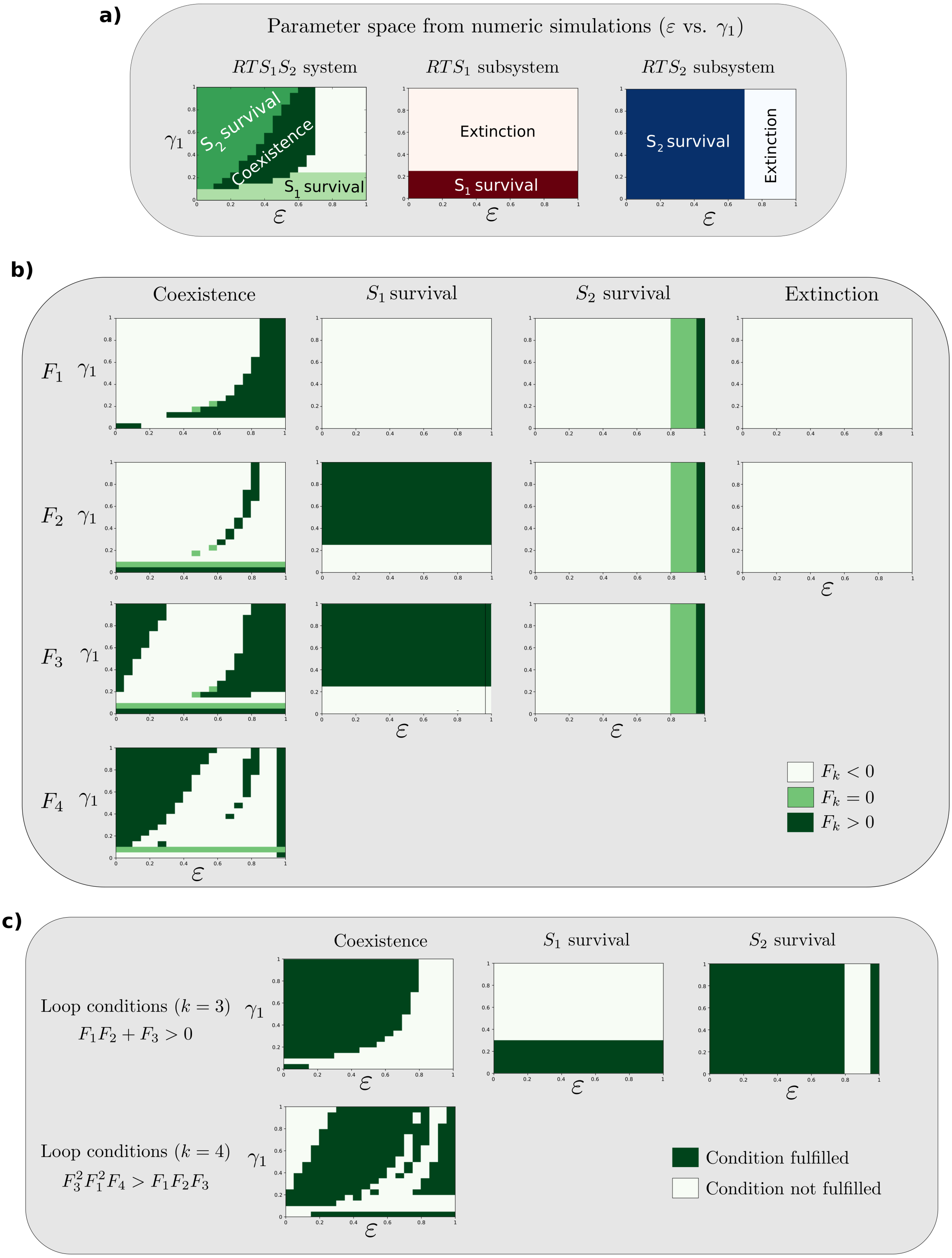

FIG. 10: Parameter space ε vs γ_1. Crosscheck between numerical results and qualitative stability analysis using the Loop Analysis method. **a)** Parameter space obtained from numerical simulations. Three initial conditions are shown, from left to right: both species present at $t = 0$, only S_1 present at $t = 0$ and only S_2 present at $t = 0$. **b)** Checking the sign of each feedback at level k (F_k's, from $k = 1...4$), to check if condition 1 section VI-B (see text) is fulfilled. **c)** Checking if the loop overwhelming conditions are fulfilled (see condition 2 section VI-B in text).

2. Longer loops **must not overwhelm the strength** of shorter loops. This condition is explicitly defined through

Routh-Hurwitz criteria, having the equivalence between F_k's and the coefficients of the characteristic equation polynomial. Importantly, the check if this conditions holds you obviously need to know the magnitudes and fixed points of your system.

Let us explicit the 2nd condition, which is rooted in Routh-Hurwitz criteria. Being $\mathbf{A}$ a $m \times m$ matrix, the characteristic equation is of the form

$$\lambda^m + a_1\lambda^{m-1} + a_2\lambda^{m-2} + ... + a_m = 0 \tag{21}$$

The Routh-Hurwitz criterion gives constraints on the coefficients $a_1...a_m$ which are necessary and sufficient to ensure all eigenvalues lie in the left half complex plane[5]. Concerning our analysis, the conditions of interest are the ones for $m = 3$ and 4:

$$a_1a_2 > a_3 \ (m = 3)$$
$$a_1a_2a_3 > a_3^2a_1^2a_4 \ (m = 4)$$

We refer the reader to Levins 1975[3] to assess why the equivalence $a_k = -F_k$ is correct. This equivalence allows us to write the following inequalities in F_k's terms:

$$F_1F_2 + F_3 > 0 \ (k = 3) \tag{22}$$
$$F_3^2F_1^2F_4 > F_1F_2F_3 \ (k = 4) \tag{23}$$

From these basic concepts we can already define the F_k's of our system, and together with inequalities 22 and 23 gain insight into its stability, which we will be able to crosscheck with the numerical results presented in the paper. Let us develop the expressions for $F_{k=1...4}$ below using equation 20:

$$\begin{aligned} F_1 &= (R) + (T) \\ &= -a_{RR} - a_{TT} < 0 \end{aligned}$$

therefore, F_1 will always be negative.

$$\begin{aligned} F_2 &= (TS_2) + (RS_1) + (RS_2) - (R)(T) \\ &= (-a_{S_2T})(-a_{TS_2}) + (a_{S_1R})(-a_{RS_1}) + (a_{S_2R})(-a_{RS_2}) - (-a_{RR})(-a_{TT}) \\ &= a_{S_2T}a_{TS_2} - a_{S_1R}a_{RS_1} - a_{S_2R}a_{RS_2} - a_{RR}a_{TT} < 0 \end{aligned}$$

in the case of F_2 we are left with the inequality

$$a_{S_2T}a_{TS_2} < a_{S_1R}a_{RS_1} + a_{S_2R}a_{RS_2} + a_{RR}a_{TT} \tag{24}$$

which must be fulfilled in order to have stability of our system (around the coexistence equilibrium point $\mathbf{P_2}$ defined in section **S-1**).

$$\begin{aligned} F_3 &= -(R)(TS_2) - (T)(RS_1) - (T)(RS_2) \\ &= -(-a_{RR})(-a_{S_2T})(-a_{TS_2}) - (-a_{TT})(a_{S_1R})(-a_{RS_1}) - (-a_{TT})(a_{S_2R})(-a_{RS_2}) \\ &= a_{RR}a_{S_2T}a_{TS_2} - a_{TT}a_{S_1R}a_{RS_1} - a_{TT}a_{S_2R}a_{RS_2} < 0 \end{aligned}$$

again, in this case for F_3 we are left with another inequality

$$a_{RR}a_{S_2T}a_{TS_2} < a_{TT}a_{S_1R}a_{RS_1} + a_{TT}a_{S_2R}a_{RS_2} \tag{25}$$

which again must be fulfilled to ensure the system's stability.
Finally, F_4 is always negative:

$$\begin{aligned} F_4 &= (S_2TS_1R) \\ &= (-a_{TS_2})(-a_{S_1T})(-a_{RS_1})(a_{S_2R}) \\ &= -a_{TS_2}a_{S_1T}a_{RS_1}a_{S_2R} < 0 \end{aligned}$$

The inequalities of F_2 and F_3 indicate that our system's stability is conditionally stable to the magnitudes of its interactions, but once the inequalities hold, the only requirements for the system's stability are the ones defined in eqns. 22 and 23.

As we have our system of equations defined, we know the expression for the entries a_{ij} of our system's community matrix $\mathcal{J}$ defined in **S-I**. Substituting the actual a_{ij} to the F_k's expressions we obtain

$$F_1 = -\delta_R - \eta(S_1 + S_2) - \delta_T - \varepsilon S_2 \tag{26}$$
$$F_2 = \gamma_2 S_2 \varepsilon T < \rho S_1 \eta R + \rho(1-\varepsilon) S_2 \eta R + (\delta_R + \eta(S_1+S_2))(\delta_T + \varepsilon S_2) \tag{27}$$
$$F_3 = [\delta_R + \eta(S_1+S_2)](\gamma_2 \varepsilon S_2 T) < (\delta_T + \varepsilon S_2)(\eta \rho R S_1) + (\delta_T - \varepsilon S_2)(\eta\rho(1-\varepsilon) R S_2) \tag{28}$$
$$F_4 = -\varepsilon T \gamma_1 S_1 \eta R \rho (1-\varepsilon) S_2 \tag{29}$$

Note that to obtain the full expressions of $F_{k=1\ldots4}$ in terms of the parameters we would need to substitute the fixed points into the above expressions. These expressions work for all the fixed points of the system: the only difference is that in the subsystems $\mathbf{RTS_1}$ and $\mathbf{RTS_2}$, F_4 would be equal to zero, as no loops of length 4 are present (and no combinations of disjunct loops of $k = 4$ species are possible). The simplest subsystem would be a **RT** system, in which F_1 and F_2 would always be negative and F_3 and F_4 would be equal to zero, for the same reason as the subsystems of three elements.

The conditions of eqns. 22 and 23 are not expressed in terms of the system's constants as they would be too involved.

Crosscheck of numeric simulations with qualitative stability conditions

In order to validate our numerical simulations we have checked if the values of $F_{k=1\ldots4}$ are negative and the loop overwhelming conditions (eqns. 22 and 23) hold under the conditions tested in the main text. The results can be checked in Fig. 8 and 7 for the μ_T vs. μ_R parameter spaces (conditions $\gamma_1/\gamma_2 \gg 1$ and $\gamma_1/\gamma_2 > 1$, respectively) and in Fig. 9 for the parameter space of ε vs. γ_1. The results of the numerical simulations, for the complete system $\mathbf{RTS_1S_2}$ and the subsystems RTS_1 and RTS_2 are also included in the mentioned figures.

VII. COLLAPSE OF PARAMETERS

The 4D system presented in the main text can have four parameters collapsed into two without affecting the properties of interest in this paper, namely δ_R and δ_T can be collapsed to δ, while δ_1 and δ_2 can be collapsed into δ_S. This means that the two species don't need to be asymmetric in all the parameters for the coexistence to exist.

The system then reads

$$\dot{T} = \mu_T - \delta T - \varepsilon S_2 T \tag{30}$$
$$\dot{R} = \mu_R - \delta R - \eta(S_1 + S_2) R \tag{31}$$
$$\dot{S_1} = \rho R S_1 - \gamma_1 T S_1 - \delta_S S_1 \tag{32}$$
$$\dot{S_2} = \rho(1-\varepsilon) R S_2 - \gamma_2 T S_2 - \delta_S S_2 \tag{33}$$

and the corresponding fixed points:

$$FP_1 = \left(\frac{\mu_T}{\delta}, \frac{\mu_R}{\delta}, 0, 0\right)$$
$$FP_2 = \left(\frac{\delta_S[(1-\varepsilon)-1]}{\gamma_2 - \gamma_1(1-\varepsilon)}, \frac{\delta_S(\gamma_2-\gamma_1)}{\rho(\gamma_2 - \gamma_1(1-\varepsilon))}, [\gamma_2 - \gamma_1(1-\varepsilon)]\left[\frac{\mu_R \rho}{\eta \delta_S(\gamma_2-\gamma_1)} - \frac{\mu_T}{\varepsilon \delta_S[(1-\varepsilon)-1]}\right] + \delta\left(\frac{1}{\varepsilon} - \frac{1}{\eta}\right), -\frac{\mu_T[\gamma_2-\gamma_1(1-\varepsilon)]}{\varepsilon^2 \delta_S} - \frac{\delta}{\varepsilon}\right)$$
$$FP_3 = \left(\frac{\mu_T}{\delta}, \frac{\mu_T\gamma_1 + \delta_S\delta}{\rho\delta}, \frac{\rho\mu_R\delta - \mu_T\gamma_1\delta - \delta_S\delta^2}{\eta(\mu_T\gamma_1 + \delta_S\delta)}, 0\right)$$
$$FP_{4,5} = \left(\frac{\mu_T}{\delta + \varepsilon S_2^*}, \frac{\mu_R}{\delta + \eta S_2^*}, 0, S_2^*\right)$$

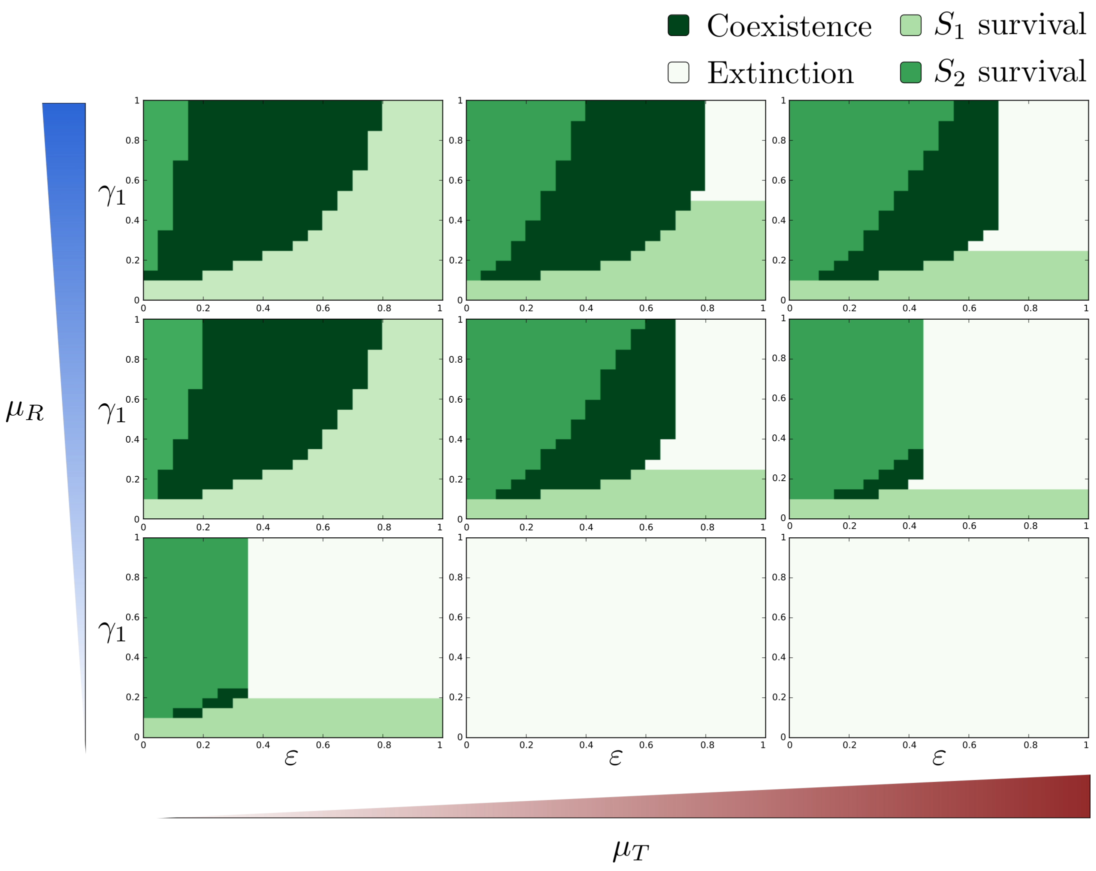

FIG. 11: Parameter space ε vs. γ_1 taking equidistant conditions of μ_R and μ_T (the values of the two last constants are indicated as gradients). We observe how ε and γ_1 are positively correlated regarthless of the values of μ_R and μ_T.

with

$$S_2^* = -\frac{1}{2\delta_S \varepsilon \eta}\Big[\delta\delta_S\varepsilon + \delta\delta_S\eta + \eta\gamma_2\mu_T - \varepsilon\mu_R\rho + \varepsilon^2\mu_R\rho \pm \Big\{4\delta_S\varepsilon\eta\left(-\delta^2\delta_S - \delta\gamma_2\mu_T + \delta\mu_R\rho - \delta\varepsilon\mu_R\rho\right) + \left[-\delta\delta_S\varepsilon - \delta\delta_S\eta - \eta\gamma_2\mu_T + \varepsilon\mu_R\rho - \varepsilon^2\mu_R\rho\right]^2\Big\}^{1/2}\Big]$$

VIII. CORRELATION OF INDIRECT POSITIVE LINK FROM S_2 TO S_1 MAINTAINED UNDER DIFFERENT CONDITIONS

The relationship between γ_1 and ε shown in the main text (Fig. 3) is maintained for different values of μ_R and μ_T, as can be observed in Fig. 10.

IX. HOPF BIFURCATION

If we explore other parameter regions that the ones shown in the main text for the 4D system, a subcritical Hopf bifurcation is encountered in a particular region of the parameter space (see Fig. 11).

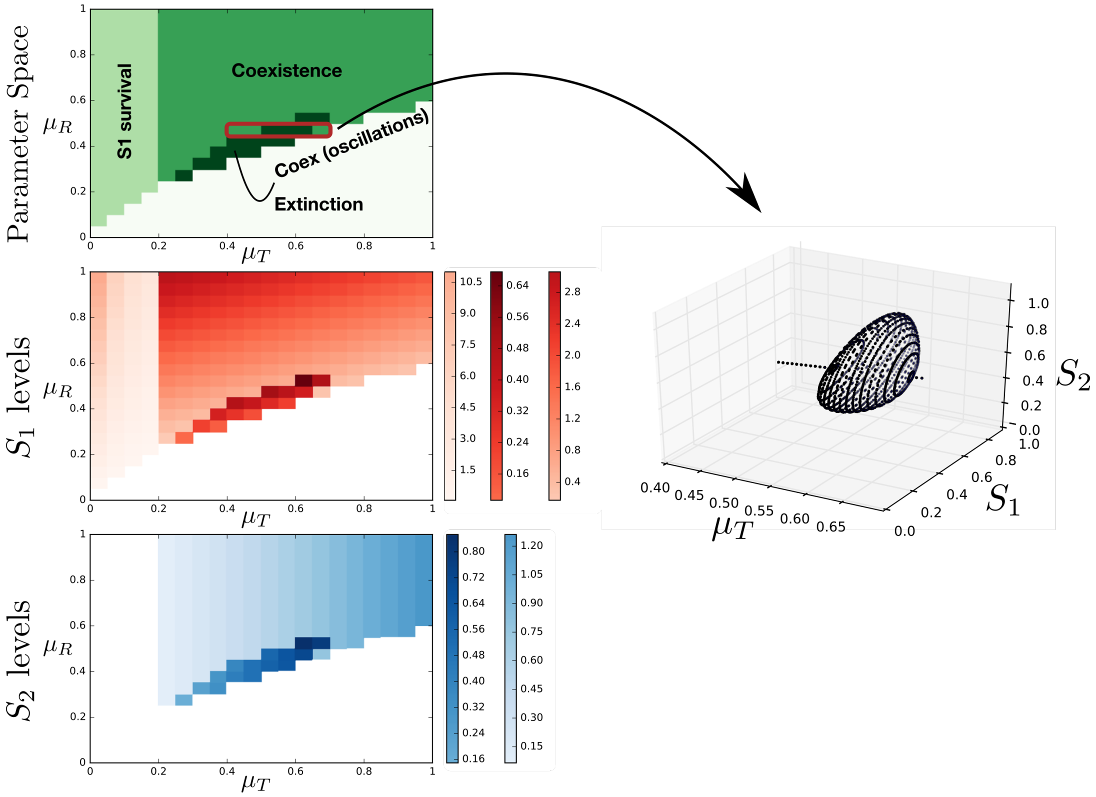

FIG. 12: Hopf bifurcation. The parameters used in this case are: $\gamma_1/\gamma_2 \gg 1$, . $\rho = 0.3$, $\varepsilon = 0.3$, $\eta = 0.1$, $\delta_R = \delta_T = 0.1$, $\gamma_1 = 0.3$, $\gamma_2 = 0.1$, $\delta_1 = 0.1$ and $\delta_2 = 0.3$.

X. DISCRETE STOCHASTIC MODEL

The discrete stochastic model follows a set of basic rules sketched in Fig. 12. Each position (i, j) in the lattice contains discrete values of N_T, N_R, N_{S_1} (*reactants*) and N_{S_2}, and they will react locally with the reactants located in the same position (i, j). In this model there are up to three different types of reactions, as we observe in Fig. 12:

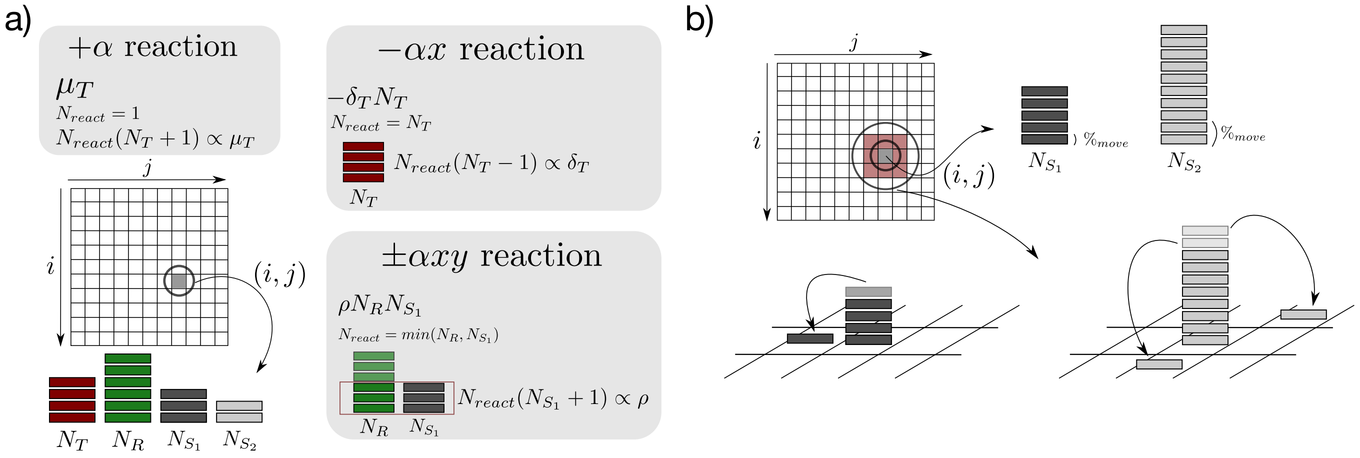

FIG. 13: Schematic representation of the spatial stochastic implementation.

i) $+\alpha$ *reaction*: just N_T and N_R contain this type of reaction, namely the influx of these substances that occurs just once ($N_{react} = 1$) for each position (i, j) in the lattice.

ii) $-\alpha x$ *reaction*: all the dimensions of the system contain this type of reaction ("one-species" reaction), namely

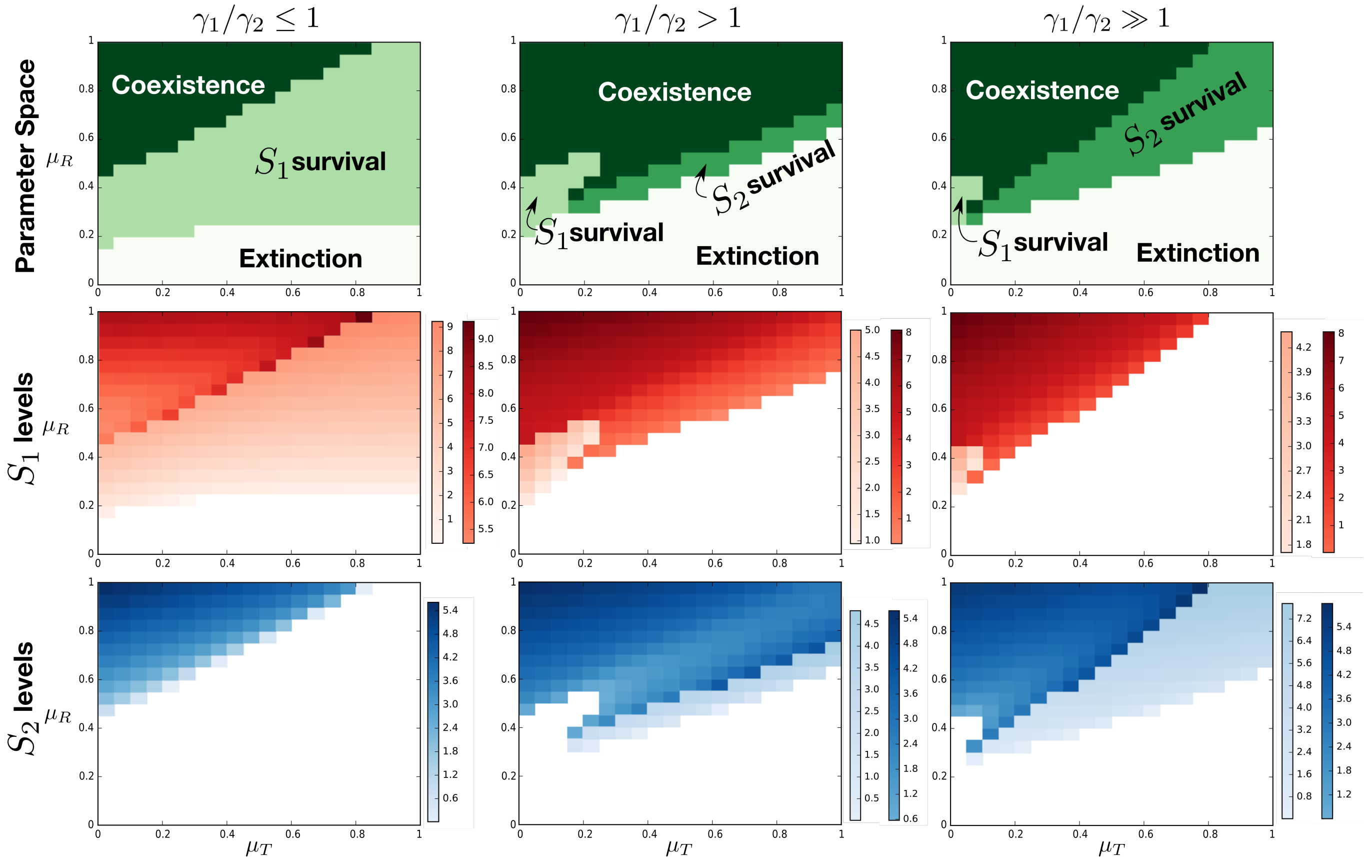

FIG. 14: **Parameter space for the discrete stochastic model under different conditions of γ_1/γ_2 (each column) as a function of μ_T and μ_R.** We observe how the results observed for the numerical simulations in Fig. **??** are qualitatively recovered, while some changes in the steady state are achieved with this stochastic counterpart in some regions of the parameter space. Here we also show the levels of S_1 and S_2 in the second and third rows, respectively (added information to Figure 4 of main text). For $\gamma_1/\gamma_2 \leq 1$, $\gamma_1 = \gamma_2 = 0.1$, for $\gamma_1/\gamma_2 > 1$, $\gamma_1 = 0.17$ and $\gamma_2 = 0.1$ and for $\gamma_1/\gamma_2 \gg 1$, $\gamma_1 = 0.3$ and $\gamma_2 = 0.1$

the natural degradation of R and T and the natural death of S_1 and S_2. In this case, the number of reactions coincide with the levels of each dimension ($N_{react} = N_x$), and each reaction will take place independently with probability α.

iii) $\pm\alpha xy$ *reaction*: again all the dimensions of the system contain this type of reaction ("two-species" reaction), namely the active decay of R and T due to one or both of the species, the growth term of S_1 and S_2, and the toxic-caused death of both species. In this case, we assume that the number of reactions that will take place is equal to the minimum value among the two reactants ($N_{react} = min(x, y)$), and again this reaction will take place independently with probability α.

We also schematically represent the random movement pursued by reactants S_1 and S_2 in Fig. S12b. In Fig. 13 we get some extra information with respect to Fig. 4 of the main text, as the levels of S_1 and S_2 species are included.

A. Maintenance of the positive correlation of indirect positive link parameter values

The result regarding the positive relation between γ_1 and ε, namely the parameters conforming the indirect positive link, observed in Fig. 3 of the main text is maintained in the spatial stochastic version of the model. This result can be observed in Fig. 14.

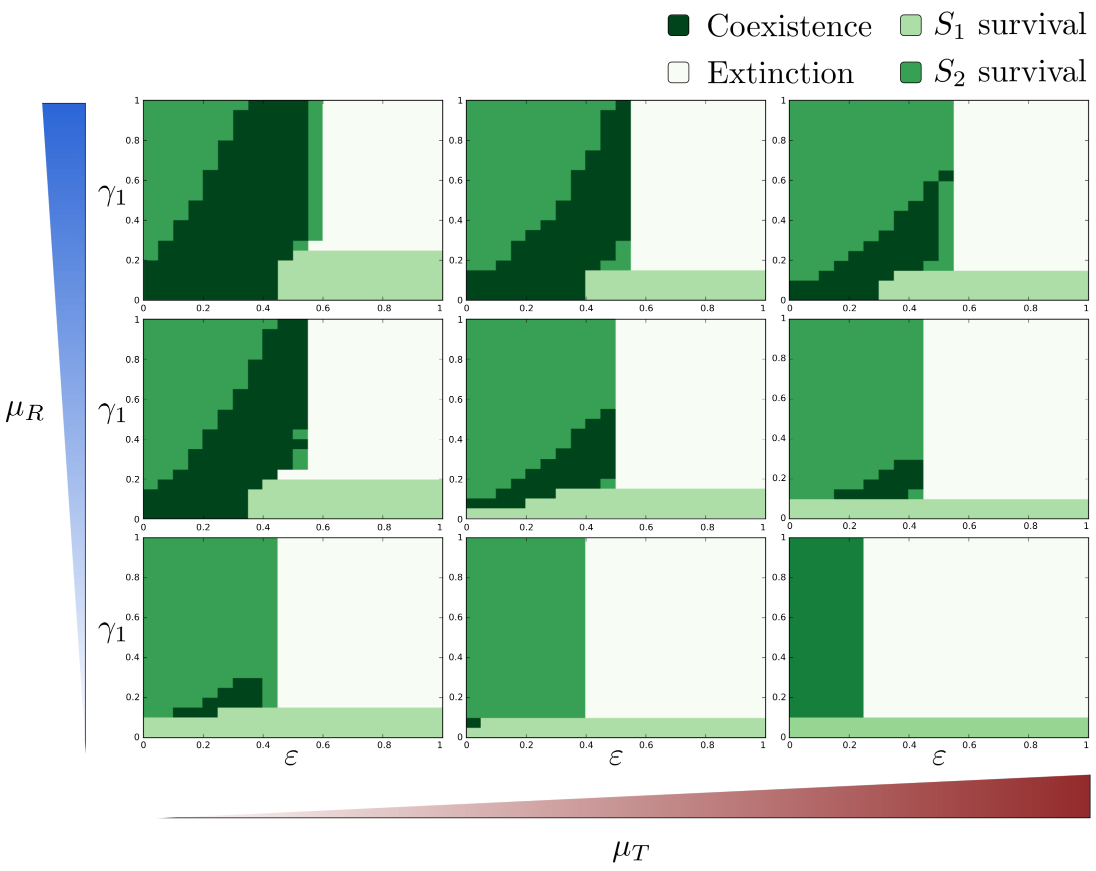

FIG. 15: Stochastic Spatial Model: Parameter space ε vs. γ_1 taking equidistant conditions of μ_R and μ_T (the values of the two last constants are indicated as gradients). We observe how ε and γ_1 are positively correlated regarthless of the values of μ_R and μ_T.

B. Temporal evolution of R, T, S_1 and S_2

The temporal evolution that follow the four reactants of the Discrete Stochastic model for a set of selected conditions can be observed in Fig. 15. As it can be checked in the figure, the reactants rapidly reach its stable stable where they slightly fluctuate around it.

XI. NUMERICAL SIMULATIONS

The numerical simulations have been done using the classical Runge-Kutta method, $\Delta t = 0.001$ and the number of iterations adapted so that the equilibrium is reached.

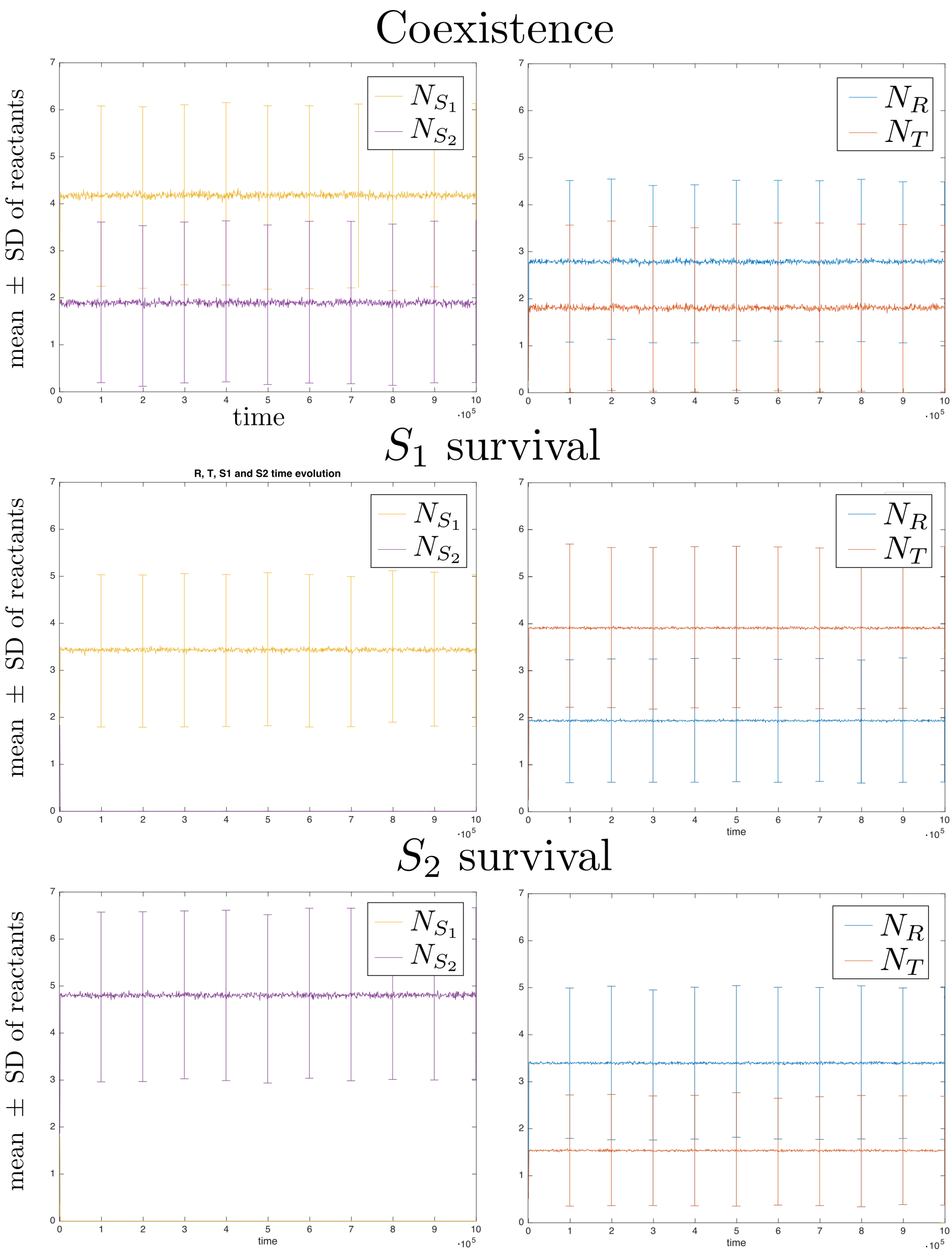

FIG. 16: Stochastic Spatial Model: Temporal evolution of the reactants (N_T, N_R, N_{S_1} and N_{S_2}), taking a sample of a situation with Coexistence ($\mu_T = 0.4$, $\mu_R = 0.7$, $\gamma_1 = 0.17$), S_1 survival ($\mu_T = 0.4$, $\mu_R = 0.4$, $\gamma_1 = 0.05$) and S_2 survival ($\mu_T = 0.8$, $\mu_R = 0.7$, $\gamma_1 = 0.3$). The rest of parameters are the same for the three cases ($\rho = 0.3$, $\varepsilon = 0.3$, $\gamma_2 = 0.1$, $\delta_R = \delta_T = \delta_1 = \delta_2 = 0.1$).

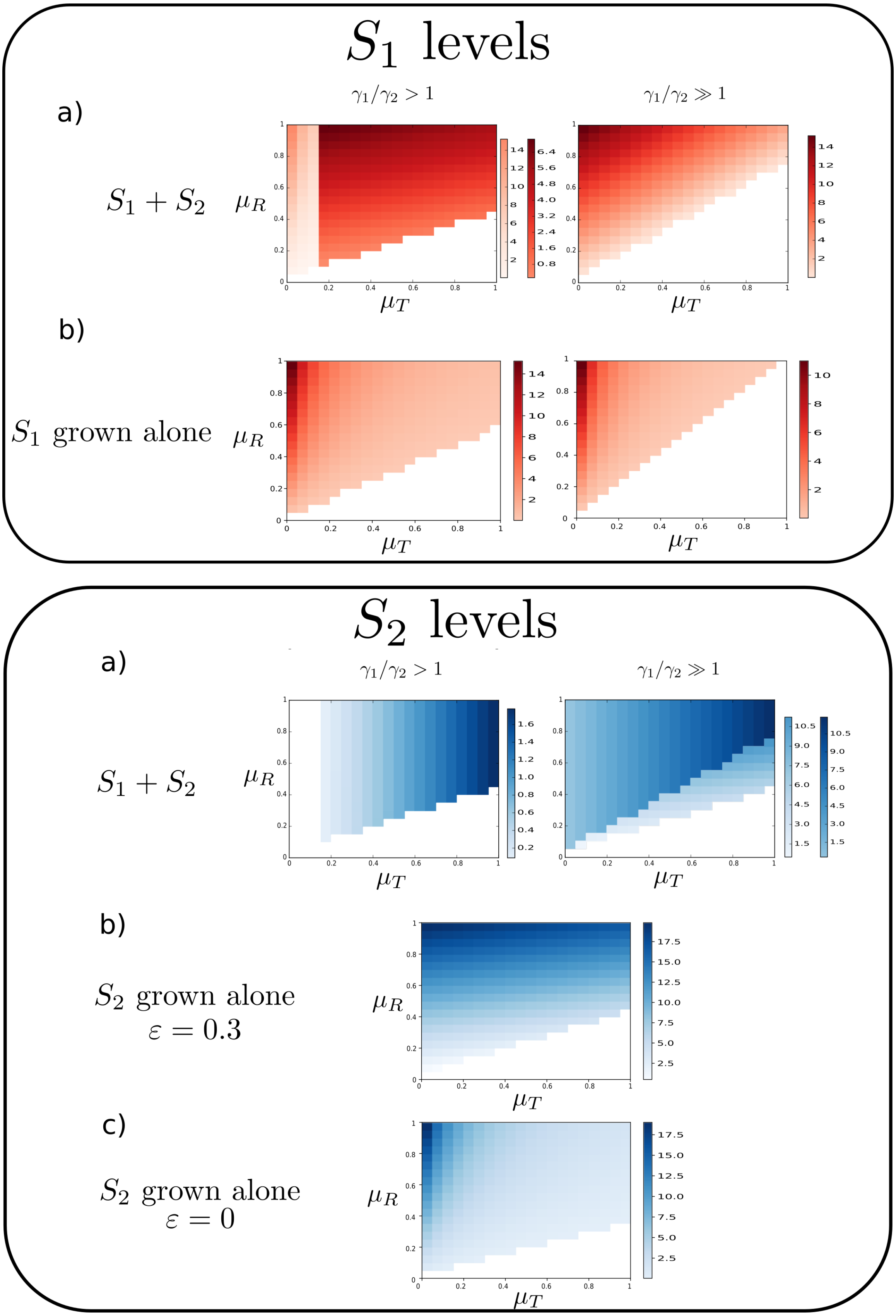

FIG. 17: S_1 **(red) and** S_2 **(blue) levels when grown together or on its own**. S_1 levels subfigure: a) S_1 levels when grown together with S_2, taken from Fig. 2 in main text. Different gradient scales in the same $\mu_R - \mu_T$ space indicates a change in the stable state of the system (check Fig. 2 main text). b) Levels of S_1 when grown alone, exposed to the same conditions as in a).S_2 levels subfigure: a) S_2 levels when grown together with S_1, taken from Fig. 2 in main text. Different gradients scales in the same $\mu_R - \mu_T$ space indicates a change in the stable state of the system (check Fig. 2 main text). b) Levels of S_2 when grown alone, exposed to the same conditions as in a) -maintaining $\varepsilon = 0.3$. c) Equivalent to b), but now with $\varepsilon = 0$.

XII. NATURE OF THE SOCIAL INTERACTION BETWEEN S_1 AND S_2

A. $\mu_R - \mu_T$ parameter space

The levels of S_1 and S_2 have been assessed when grown on its own or together in equivalent μ_R and μ_T conditions (see Fig. 16). We clearly observe how the addition of S_2 allows S_1 to grow in regions that were forbidden for it when grown alone (see Fig. 16 (S_1 levels subfigure)). It is also observed how the fitness of S_1 increases, reaching higher levels, when coexisting with S_2, confirming the exploitation of the cooperative trait of S_2 by S_1. This happens for all the tested values. On the other hand, in S_2 levels subfigure of Fig. 16, comparing c) with b) we can assess that, for increasing values of μ_T, the payoff incurred by increasing ε in reproductive capacity is completely outweighted by the advantages it entails the detoxifying capacity, as we observe higher fitness of S_2 in b) than in c). Only in some residual cases, for μ_T too low, we can find that the payoff of the cooperative trait does not outweight its costs. Comparing b) with a), we can observe how the exploitation of the cooperative trait by S_1 clearly diminishes the fitness of S_2 when grown together. For the condition where $\gamma_1/\gamma_2 > 1$, the maximum fitness of S_2 decreases from ~ 18 to ~ 1.7, while for $\gamma_1/\gamma_2 \gg 1$, the decrease is not so dramatic due to the higher toxic penalization to S_1, but still the maximum fitness of S_2 decreases to ~ 11.

Comparing one by one the levels of S_2 with $\varepsilon = 0$ and the ones where coexistence is present (with $\varepsilon = 0.3$), we can clearly find two interesting phases (see Fig. 17). Following the definition in [11], we assign Altruistic behaviour to the cases in which the fitness of S_2 when coexisting with S_1 -while displaying its cooperative trait-, is lower compared to its fitness when $\varepsilon = 0$, that is, when no energy allocation was invested in any cooperative trait. This cases show high exploitation by S_1 of the cooperative trait. On the other hand, 'Mutually beneficial' is assigned when, despite the exploitation of S_1, the cooperative species S_2 still has higher fitness than the situation in which $\varepsilon = 0$. Interestingly, the mutual benefit of the cooperative trait occurs with a positive correlation of μ_T and μ_R.

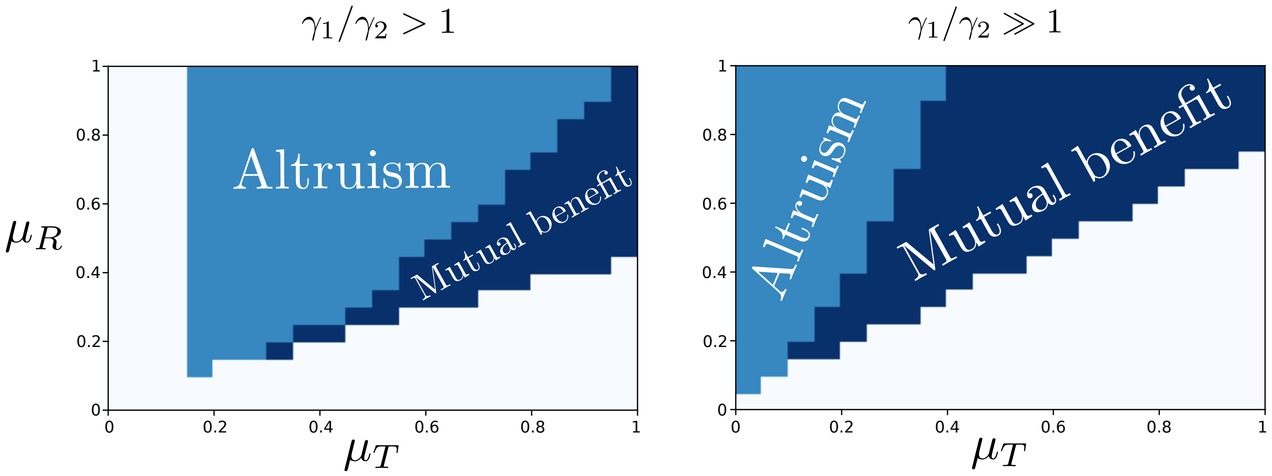

FIG. 18: **Social interaction behind the cooperative trait of S_2.** We observe how the cooperative trait can be both altruistic and mutually beneficial, taking the definition from [11]. At left, we observe the phases for $\gamma_1/\gamma_2 > 1$, at right, for $\gamma_1/\gamma_2 \gg 1$. For the latter case, the 'mutual benefitial' region expands

B. $\varepsilon - \gamma_1$ parameter space

We have also assessed the effect of the ε parameter value (balancing both the detoxifying effect of S_2 and its diminished reproductive capacity as a metabolic tradeoff) to see its effects in the exploitation capacity of S_1. In Fig. 18, we observe how the detoxifying effect of S_2 (proportional to ε) is clearly a cooperative trait which benefits itself compared to the basal phenotype -S_2 with $\varepsilon = 0$- (check that the fitness of S_2 is maximal at $\varepsilon \sim 0.2$ despite implying a loose in reproduction capacity proportional to $(1 - \varepsilon)$) but that is rapidly exploited by S_1 once ε reaches a certain threshold, allowing this species to reach a much higher fitness peak compared to the maximum it can reach in the absence of the cooperative species S_2 (check Fig. 18a, left plot, where S_1 survival on its own is observed). Importantly, Fig. 18a does not evaluate $\varepsilon = 0$, but it is evaluated in Fig. 18b, where we see that its fitness decreases

even more. This value is the one we use to assess if we observe a cooperative trait which is mutually beneficial or altruistic.

Concretely, the fitness of S_2 with $\varepsilon = 0$ is 1.5 (in this particular parameter conditions, with $\mu_R = \mu_T = 0.5$. The rest of the parameters are included in the caption of Fig. 3 in main text). When grown alone, above $\sim \varepsilon = 0.72$, the cooperative trait does not benefit its own costs anymore, so it would not be likely to observe a feature like this in nature. When grown together with S_1, with this species exploiting the cooperative trait, we observe how for $\gamma_1 = 0.2$, the cooperative trait transitions from being mutually beneficial to being altruistic (as the fitness of S_2 goes below 1.5) at $\varepsilon \sim 0.32$, for $\gamma_1 = 0.5$ the transition occurs at $\varepsilon \sim 0.57$ while for $\gamma_1 = 0.9$, it occurs at $\varepsilon \sim 0.71$.

Therefore, using these tests we have observed that the cooperative trait of S_2 can transition from mutually beneficial to altruistic. However, it is also clear that when this transition occurs the levels of both S_1 and S_2 are already considerably low. These results, taking together with the previous analysis of the parameter space $\mu_R - \mu_T$ shows how all these parameters can highly influence the final outcome of the social interaction between the competitors S_1 and S_2.

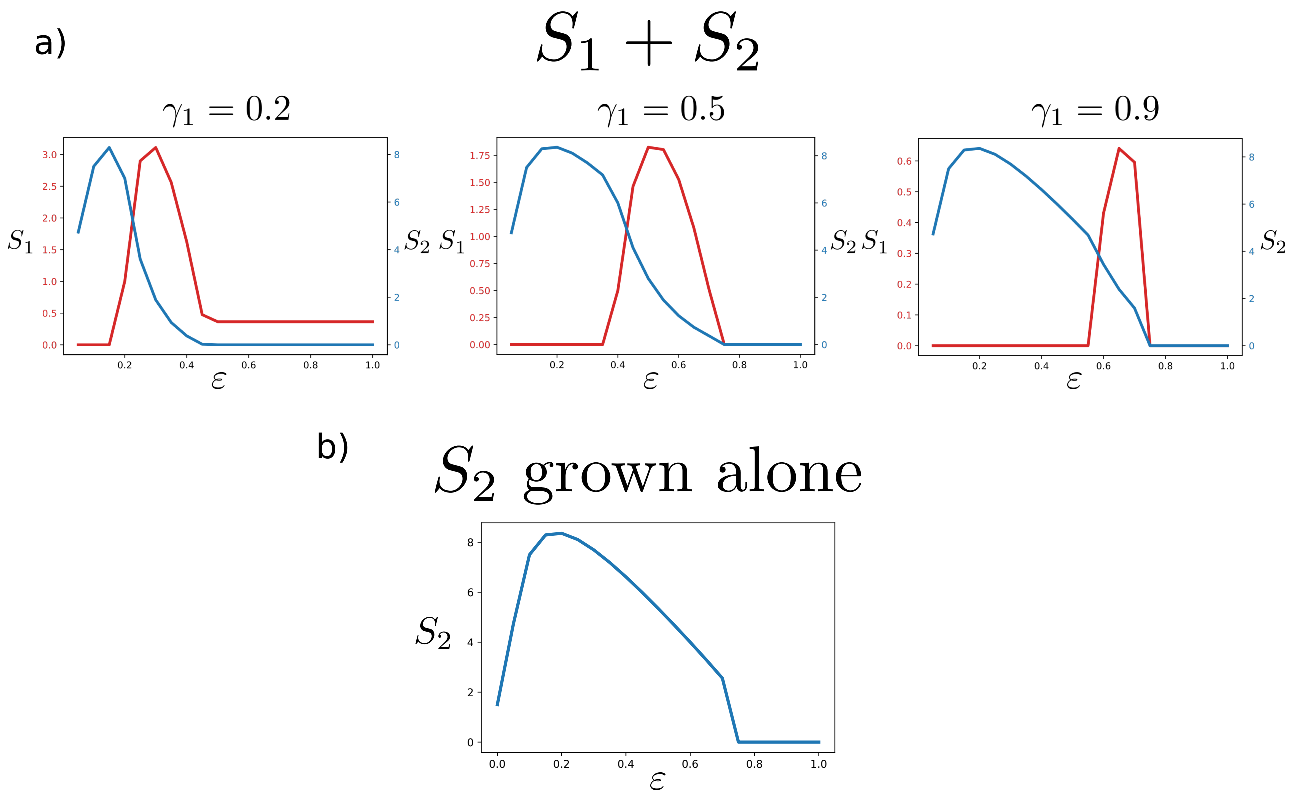

FIG. 19: **S_1 and S_2 levels grown together or on its own for varying values of ε and fixed γ_1.** a) Plots taken from Fig. 3c of main text, to provide a better comparison with b), where we show the levels of S_2 when grown alone for varying values of ε.

XIII. EFFECT OF γ_2 ON THE NONLINEAR BOUNDARY DELIMITING COEXISTENCE IN THE ε-γ_1 PARAMETER SPACE

In the main text we derived that the relationship between ε and γ_1 followed a nonlinear positive correlation (see Section III- *Positive correlation of indirect positive link parameter values*). However, we found also important not to neglect γ_2 from that relationship, as it establishes the boundary below which coexistence is not possible (see inequality 18 from main text). Here we have plot the inequality 18 (see main text) above the parameter spaces $\varepsilon - \gamma_1$ to see how it perfectly matches the boundary between coexistence and S_2 and, besides, the clear effect γ_2 exerts (see Fig. 19).

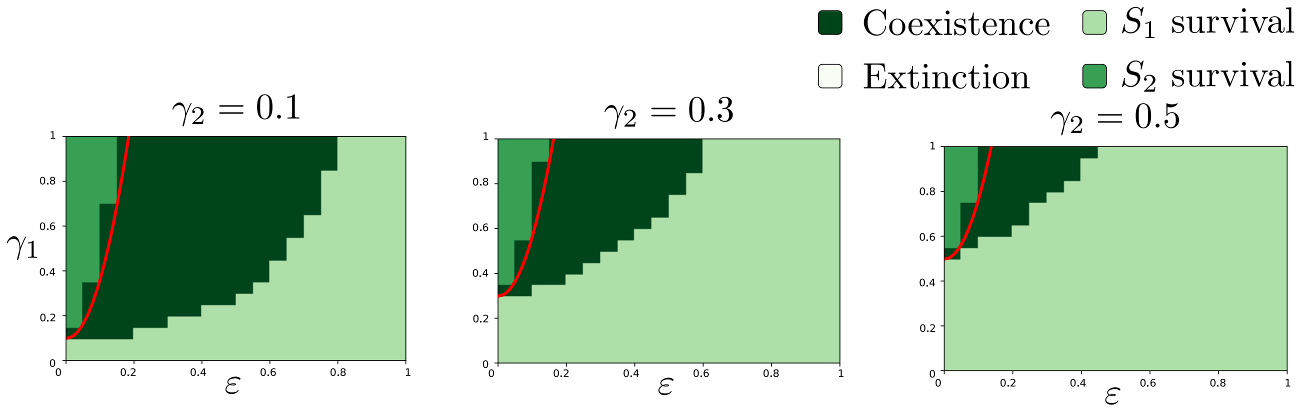

FIG. 20: **Effect of γ_2 on the positive nonlinear correlation between ε and γ_1.**. The parameter spaces formed by ε and γ_1 with the different phases (coexistence, S_1/S_2 survival or extinction) are shown. From left to right, the effect of increasing values of γ_2 is observed, changing the region where coexistence is possible. The values for the rest of the parameters are: $\mu_R = 0.9$, $\mu_T = 0.1$, $\delta_1 = \delta_2 = 0.1$, $\rho = 0.3$, $\eta = 0.1$, $\delta_R = \delta_T = 0.1$.

XIV. REFERENCES

[1] Edelstein-Keshet L. 1988 *Mathematical models in biology* vol. 46. Siam.

[2] Levins R. 1974 Discussion paper: the qualitative analysis of partially specified systems. *Annals of the New York Academy of Sciences* **231**, 123–138.

[3] Levins R. 1975 Evolution in communities near equilibrium. *Ecology and evolution of communities* pp. 16–50.

[4] May RM. 1973 Qualitative stability in model ecosystems. *Ecology* **54**, 638–641.

[5] May RM. 2001 *Stability and complexity in model ecosystems* vol. 6. Princeton university press.

[6] Quirk J, Ruppert R. 1965 Qualitative economics and the stability of equilibrium. *The review of economic studies* **32**, 311–326.

[7] Jeffries C. 1974 Qualitative stability and digraphs in model ecosystems. *Ecology* **55**, 1415–1419.

[8] Dambacher JM, Luh HK, Li HW, Rossignol PA. 2003 Qualitative stability and ambiguity in model ecosystems. *The American Naturalist* **161**, 876–888.

[9] Mason SJ. 1953 Feedback theory. .

[10] Wright S. 1921 Correlation and causation. *Journal of agricultural research* **20**, 557–585.

[11] West SA, Griffin AS, Gardner A, Diggle SP. 2006 Social evolution theory for microorganisms. *Nature reviews microbiology* **4**, 597.
